# Synthetic high-density lipoprotein nanoparticles for the treatment of Niemann–Pick diseases

**DOI:** 10.1186/s12916-019-1423-5

**Published:** 2019-11-11

**Authors:** Mark L. Schultz, Maria V. Fawaz, Ruth D. Azaria, Todd C. Hollon, Elaine A. Liu, Thaddeus J. Kunkel, Troy A. Halseth, Kelsey L. Krus, Ran Ming, Emily E. Morin, Hayley S. McLoughlin, David D. Bushart, Henry L. Paulson, Vikram G. Shakkottai, Daniel A. Orringer, Anna S. Schwendeman, Andrew P. Lieberman

**Affiliations:** 1 Department of Pathology, University of Michigan Medical School, 3510 MSRB1, 1150 W. Medical Center Dr., Ann Arbor, MI 48109 USA; 2 Department of Medicinal Chemistry, College of Pharmacy, University of Michigan, Ann Arbor, MI 48109 USA; 3 Department of Neurosurgery, University of Michigan Medical School, Ann Arbor, MI 48109 USA; 4 Cellular and Molecular Biology Graduate Program, University of Michigan Medical School, Ann Arbor, MI 48109 USA; 5 Medical Scientist Training Program, University of Michigan Medical School, Ann Arbor, MI 48109 USA; 6 Department of Pharmaceutical Sciences, University of Michigan College of Pharmacy, B20-102W NCRC, 2800 Plymouth Road, Ann Arbor, MI 48109 USA; 7 Department of Neurology, University of Michigan Medical School, Ann Arbor, MI 48109 USA; 8 Department of Molecular & Integrative Physiology, University of Michigan Medical School, Ann Arbor, MI 48109 USA; 9 Biointerfaces Institute, University of Michigan, Ann Arbor, MI 48109 USA

**Keywords:** Niemann–Pick C, NPC1, HDL, SRS, Niemann–Pick A

## Abstract

**Background:**

Niemann–Pick disease type C is a fatal and progressive neurodegenerative disorder characterized by the accumulation of unesterified cholesterol in late endosomes and lysosomes. We sought to develop new therapeutics for this disorder by harnessing the body’s endogenous cholesterol scavenging particle, high-density lipoprotein (HDL).

**Methods:**

Here we design, optimize, and define the mechanism of action of synthetic HDL (sHDL) nanoparticles.

**Results:**

We demonstrate a dose-dependent rescue of cholesterol storage that is sensitive to sHDL lipid and peptide composition, enabling the identification of compounds with a range of therapeutic potency. Peripheral administration of sHDL to *Npc1 I1061T* homozygous mice mobilizes cholesterol, reduces serum bilirubin, reduces liver macrophage size, and corrects body weight deficits. Additionally, a single intraventricular injection into adult *Npc1 I1061T* brains significantly reduces cholesterol storage in Purkinje neurons. Since endogenous HDL is also a carrier of sphingomyelin, we tested the same sHDL formulation in the sphingomyelin storage disease Niemann–Pick type A. Utilizing stimulated Raman scattering microscopy to detect endogenous unlabeled lipids, we show significant rescue of Niemann–Pick type A lipid storage.

**Conclusions:**

Together, our data establish that sHDL nanoparticles are a potential new therapeutic avenue for Niemann–Pick diseases.

## Background

Niemann–Pick disease type C is a fatal lysosomal storage disorder that causes progressive neurodegeneration along with visceral organ involvement. Symptom onset and disease severity are variable, but patients commonly develop hepatosplenomegaly, cognitive decline, and seizures, culminating in death in the second or third decades of life [1, 2]. Niemann–Pick C patients have loss-of-function mutations in the NPC2 (~ 5%) or, more commonly, the NPC1 (~ 95%) protein. In the late endosome/lysosomal compartment (LE/Lys), LDL-derived unesterified cholesterol is bound by NPC2 and transferred to the transmembrane NPC1 protein [3, 4]. Using a poorly defined mechanism, NPC1 exports unesterified cholesterol from LE/Lys. Unesterified cholesterol then moves to other sites within the cell where it alters membrane dynamics or is utilized for steroid production [4]. In patients with Niemann–Pick C, mutations in NPC1/NPC2 prevent intracellular lipid trafficking and cause characteristic cholesterol accumulation [5]. A biochemically similar lipid storage disease arises from mutations in the gene encoding the lysosomal enzyme acid sphingomyelinase. Deficiency of enzyme activity causes Niemann–Pick disease types A and B, in which the storage of sphingolipids and cholesterol in LE/Lys leads to hepatosplenomegaly and varying degrees of neurodegeneration [6].

Endogenous mechanisms to maintain cellular cholesterol homeostasis include the removal of excess cholesterol by high-density lipoprotein (HDL) particles. Cholesterol is effluxed from peripheral cells by nascent HDL particles and esterified in plasma. Mature HDLs then travel to the liver where cholesterol is eliminated in the bile [7]. Recent work has taken advantage of endogenous HDL function for the development of synthetic HDL (sHDL) nanoparticles as potential therapeutics for cardiovascular diseases [8–11]. These nanoparticles are composed of the HDL protein apolipoprotein A-1 (ApoA1) or ApoA1 mimetic peptides surrounding a lipid bilayer to form 10–12-nm diameter discoidal lipoprotein particles [12, 13]. Chemical synthesis of sHDL permits modifications that alter lipid and ApoA1 peptide composition and thereby impact potency, pharmacokinetics, and safety [14–17]. sHDL nanoparticles were initially designed for removal of cholesterol from lipid-laden atherosclerotic plaques. In clinical trials involving ~ 2000 cardiovascular disease patients, sHDL was safe and well-tolerated [10, 11, 18–21], and a large phase III clinical trial in 17,400 patients is currently ongoing (https://clinicaltrials.gov/ct2/show/NCT03473223).

Here, we developed and optimized a sHDL nanoparticle which significantly reduces the accumulated cholesterol in Niemann–Pick type C cells. The sHDL contains a 37-amino acid ApoA1 mimetic peptide, termed 5A, and sphingomyelin (SM). 5A-SM sHDL at 1:1.15 (wt/wt) peptide to lipid ratio is safe in primates, and with established sterile manufacturing, this sHDL is well positioned for rapid clinical translation [22, 23]. We show that 5A-SM sHDLs are non-toxic and effective at reducing cholesterol storage in Niemann–Pick C patient fibroblasts and brain slice cultures from *Npc1* mutant mice. We establish that 5A-SM requires the ATP-binding cassette transporter 1 (ABCA1) to efflux stored cholesterol. In vivo studies using *Npc1* mutant mice show evidence of target engagement and rescue of peripheral phenotypes and neuronal cholesterol storage. Furthermore, we show that 5A-SM also rescues sphingomyelin storage in Niemann–Pick type A fibroblasts. Together, these studies provide proof-of-concept data to support the therapeutic potential of sHDL for the Niemann–Pick diseases.

## Methods

### Mice

All *Npc1*-I1061T mice [24] were backcrossed to C57BL/6 (≥ 10 generations). Approximately equal numbers of males and females were used for all experiments, and littermates were used when available. Mice were randomly assigned to vehicle or experimental groups. All procedures involving mice were approved by the University of Michigan Committee on Use and Care of Animals (PRO00008133) and conducted in accordance with institutional and federal guidelines.

### Reagents

2-Hydroxypropyl-β-cyclodextrin (H-107) and amiloride (A7410) were from Sigma. EndoH (P0702) and PNGaseF (P0704) were from New England Biolabs. Dynasore (14061) was from Cayman Chemical; Human HDL (J64903) and acetylated LDL (J65029) were from Alfa Aesar. 5A peptide (DWLKAFYDKVAEKLKEAF-P-DWAKAAYDKAAEKAKEAA, 4078379) was from Bachem Americas (Torrance, CA). 22A peptide (PVLDLFRELLNELLEALKQKLK) was synthesized by Genscript (Piscataway, NJ). Lipids including egg-sphingomyelin (SM, Coastome NM-10), 1,2-dimyristoyl-*sn*-glycero-3-phosphocholine (DMPC, Coastome MC-4040), and 1-palmitoyl-2-oleoyl-*sn*-glycero-3-phosphocholine (POPC, Coastome MC-6081) were from NOF America Corporation. 1,1′-Dioctadecyl-3,3,3′,3′-tetramethylindodicarbocyanine, 4-chlorobenzenesulfonate salt (DiD, D7757) and 4-(4-(dihexadecylamino)styryl)-*N*-methylpyridinium iodide (DiA, D3883) were from Invitrogen. Cholesteryl [1,2,6,7-3H(N)] linoleate (ART 1203) and sphingomyelin [choline methyl-3H] were from American Radiolabeled Chemicals (Saint Louis, MO). *N*-[6-[(7-Nitro-2-1,3-benxoxadiazol-4-yl)amino]-sphingosine-1-phosphocholine (NBD-Sphingomyelin, 810218P) was from Avanti Polar Lipids (Alabaster, AL).

### Antibodies

(Antigen, dilution, vendor, cat. no.): NPC1, 1:500, Abcam, ab134113; Actin, 1:4000, Sigma, A544; LAMP1, 1:100, Developmental Studies Hybridoma Bank University of Iowa, H4A3; Calbindin, 1:500–1:2000, Sigma, 02724; GFAP, 1:500, Dako, z0344; IBA1, 1:250, Abcam, ab5076; NeuN, 1:500, Millipore, abn78; EEA1, 1:400, Abcam, ab2900; F4/80, 1:400, abcam, ab6640.

### sHDL synthesis

sHDL particles were prepared using a lyophilization method where peptide (5A or 22A) and lipid (SM, DMPC or POPC) were dissolved in acetic acid at 1:1.5 wt/wt ratio and then lyophilized together for 24 h. HDL was fluorescently labeled by adding 4 μg DiD or DiA per 1 mg peptide directly to the acetic acid mixture of peptide and SM. The resulting lyophilized dry pellet was re-hydrated in PBS, pH 7.4, to a final peptide concentration of 10 mg/mL, vortexed, and thermocycled 3× between 55 °C and room temperature to generate sHDL particles. pH was adjusted to 7.4 and sHDLs were sterile filtered using 0.22-μm Millipore filters. Labeling of 5A peptide in sHDL (5A-SM-DiA) with AlexaFluor 647 dye was performed using Invitrogen protein labeling kit (A20173). Purification of 5A-SM-DiA-Alexa647 post-labeling was done on the size-exclusion column supplied in the kit, and the final concentration of sHDL was determined according to the manufacturer’s instructions using plate reader measurements (SynergyTM NEO HTS Multi-Mode Microplate Reader, Bio-Tek).

### sHDL characterization

Fluorescently labeled sHDL particles were analyzed by UPLC (Waters Aquity UPLC BEH125 sec 1.7 μm, 4.6 × 150 mm column) equipped with UV (220 nm) and fluorescence detectors (ex/em 644/665 nm DiD, 456/590 nm DiA, 650/665 nm AlexaFluor647). The hydrodynamic diameters of sHDL were determined by dynamic light scattering on Zetasizer Nano ZSP, Malvern Instruments (Westborough, MA). The volume intensity average values were reported. Transmission electron microscopy images were obtained on an FEI Morgagni electron microscope run at 100 kV at a magnification of 22,000× (2.1 Å/pixel) and then recorded on a Gatan Orius charge-coupled device camera. sHDL samples (3 μL of 2 μg/ml) were adsorbed for 1 min to a glow discharged 400-mesh copper grid covered with carbon-coated collodion film (Structure Probe). The grids were washed twice and then negatively stained in 0.07% uranyl formate. 22A and 5A peptides, SM, POPC, and DMPC lipids combined at 1:0.5, 1:1, and 1:2 wt/wt ratios were described previously [14, 15].

### Cells

Cell lines were obtained from the NIGMS Human Cell Repository at the Coriell Institute for Medical Research. GM08399 was used as a control (CTRL) cell line. Niemann–Pick C cell lines with mutations in the *NPC1* gene: GM18453 (I1061T/I1061T), GM17912 (P1007A/T1036 M), and GM03123 (I1061T/P237S); Niemann–Pick A (NPA) cell line with mutation in the *SMPD1* gene GM00112 (L302P/L302P). Cells were cultured in MEM, PSG, and 20% FBS [25].

### Treatments

#### Endocytosis inhibitors

Cells were pretreated with dynasore (80 μM) or amiloride (1 mM) for 30 min. Cell culture media was replaced with fresh media containing vehicle (saline), dynasore, or amiloride along with 5A-SM-DiD for 2 h. ImageJ was used to quantify DiD label intensity inside cells.

#### sHDL in cells

Cells were plated 24 h before treatment. At the start of treatment, cell culture media was replaced with media containing vehicle or sHDL. Culture media containing vehicle or sHDL was refreshed after 24 h.

#### sHDL treatment of brain slices

Slices were treated with fresh particles/media daily at a concentration of 5 mg/ml for a period of 4 days.

### siRNA transfection

Predesigned ON-TARGETplus SMARTpools containing 4 individual siRNAs per target sequence (Dharmacon Non-targeting SMARTpool D-001810-10-05, ABAC1 L-004128-00, SR-B1 L-010592-00) were transfected using TransIT-X2® (Mirus) reagent at *t* = 0 and *t* = 24 h. Imaging or RNA analysis occurred 48 h after the first transfection [25].

### Western blot

A bullet blender (Next Advance) was used to homogenize cell lysates. Protein concentrations were normalized by DC™-protein assay (Bio-Rad), and equal amounts of protein were loaded into 4–12% gradient SDS PAGE gels (Invitrogen). After electrophoresis and transfer onto a PVDF membrane, immunoreactivity was detected by ECL (Thermo Scientific) and imaged using an iBright (Thermo Fisher Scientific). ImageJ was used to quantify band intensity [25]. For Endoglycosidase H assay, lysates were separated into three reactions containing: negative control (NT), EndoH (E) (NEB P0702L), or PNGaseF (P) (NEB P0704L) [25]. After a 3-h incubation at 37 °C, samples were loaded on SDS PAGE gels as indicated above.

### Filipin staining

After treatment, cell membranes were labeled with wheat germ agglutinin® (Thermo Fisher). Cells were fixed in 4% PFA for 20 min, washed 3× in PBS, and 1× in glycine. Unesterified cholesterol was labeled with filipin labeling solution for 2 h. Filipin labeling solution: 10% FBS + 0.4% DMSO+ 0.03 mg/ml (tissue) or 0.1 mg/ml (cells) filipin. Slides were washed 3× with PBS and mounted with ProLong® Gold (Thermo Fisher) [25].

### RT-qPCR

RNA was converted to cDNA using the High Capacity Reverse Transcription kit (Applied Biosystems 4368814). Quantitative real-time PCR (RT-qPCR) was conducted in technical triplicates using 15 ng cDNA, TaqMan™ probes (Thermo Fisher) for human *HMGCR* (Hs 00168302), *HMGCS1* (Hs 00940429), *ABCA1* (Hs 01059118), *ABCG1* (Hs 00245154), *LDLR* (Hs 01092524), *NPC1* (Hs 00264835), *SCARB1* (SR-B1) (Hs 00969821), *SREBF-2* (SREBP) Hs 01081778, *GAPDH* (loading control) (4325792), and mouse *HMGCS* (Mm 01304569). RT-qPCR was performed using an ABI 7900HT Sequence Detection System and relative expression calculated by the 2^−ΔΔCt^ method using SDS software.

### Immunofluorescence staining

Cells were washed 3× with HBSS and fixed with 4% PFA for 20 min at room temperature. Cells were washed with PBS and glycine before addition of blocking solution (0.02% saponin, 10% normal goat serum (NGS), 1% BSA) for 1 h. Slides were incubated with primary antibodies overnight at 4 °C, washed with PBS + 0.02% saponin, and incubated with secondary antibody for 1 h [25]. Slides were mounted with Vectashield + DAPI (Vector Laboratories).

For slice culture: slices were floated in HBSS+/+ in 6-well plates containing Netwell™ Inserts (Corning). Samples were fixed in 4% PFA and 0.1% Triton X-100 for 1 h, rinsed 3× in PBS, then treated for 10 min of 1.5 mg/ml glycine. After three washes in PBS, slices were blocked in PBS containing 5% NGS for 1 h at room temperature. Slices were labeled with primary antibody (diluted in blocking) overnight. The following day, slices were washed 3× in PBS and labeled with Alexa conjugated secondary (1:500) for 1 h. After 3 washes in PBS, slices were stained with filipin labeling solution for 2 h, washed 3× with PBS, and mounted in ProLong Gold (ThermoFisher) and imaged by confocal microscopy. Calbindin was used to outline Purkinje cells, and filipin intensity was calculated using ImageJ.

### Preparation of cerebellar organotypic slice cultures

Cerebellar organotypic slice cultures were prepared using 30- μm thickness sagittal brain slices [26]. Four slices per brain were used in each set of experiments, split evenly between control and experimental medium. Two slices were placed together on a cell culture insert (Millipore; 0.4-μm pore size, 30 mm diameter) which contained 1.2-ml slice culture medium (either control or experimental) and were pre-incubated at 37 °C in 95% O_2_/5% CO_2_ in a 6-well plate. Control medium contained 50% minimal essential medium with Earle’s salts, 25% horse serum, 25% Hank’s balanced salts solution, 25 mM HEPES, 2 mM l-glutamine, and 6.5 mg/ml glucose. Experimental medium was prepared by adding nanoparticles at a concentration of 5 mg/ml to the aforementioned control medium. Every 24 h, cell culture inserts were transferred to a new 6-well plate which was pre-incubated at 37 °C in 95% O_2_/5% CO_2_ with control or experimental medium, as described above. Imaging and analysis of Purkinje neuron cholesterol content was performed after 96 total hours of incubation. In all cases, wild-type and NPC samples were matched so that slices were prepared on the same day and using the same reagents.

### Stereotaxic mouse ICV bolus delivery

Stereotaxic administration of nanoparticles into the right lateral ventricle via an intracerebral ventricular (ICV) injection was performed on mice under vaporized isoflurane anesthesia according to IACUC guidelines. Six- to 7-week-old mice received a single ICV bolus injection of sHDL or vehicle using established protocols [25, 27]. Each anesthetized mouse received a small scalp incision to expose the skull, and a small burr hole was drilled relative to Bregma suture: anterior-posterior + 0.3 mm, medio-lateral − 1.0 mm. A beveled needle (7758-04, Hamilton, Reno, NV) connected to a 10-μL syringe (7653-01, Hamilton, Reno, NV) was placed dorso-ventral − 3.0 mm at a rate of 1 mm/s. A 3-min wait was allotted for the brain to seal around the needle and prevent backflow of treatment around the injection site. A total of 10 μL vehicle or sHDL at a concentration of 100 mg/ml was delivered at an infusion rate of 0.5 μL/s using an injection pump (UMC4, World Precision Instruments, Inc., Sarasota, FL). Five minutes after the infusion was completed, the needle was retracted at a rate of 1 mm/s and the incision site was sutured with synthetic non-absorbable sutures (1011209, Henry Schein, Melville, NY). Mice were recovered in a temperature-controlled environment, and following surgery, the mouse weight, grooming activity, and home cage activity were recorded for up to 7 days according to IACUC guidelines.

### Microscopy

Epifluorescence: Filipin was imaged on a Zeiss Axio Imager Z1 microscope with an automated stage. Cells were focused in the green channel (wheat germ agglutinin), and 16 tiled images were captured per experiment. Images with ≥ 90% cell confluence were quantified using NIH ImageJ software [25].

Confocal imaging of cells: Fluorescently labeled sHDL particles were imaged on a Nikon A-1 confocal microscope. Co-localization coefficients were calculated using Nikon elements software (Pearson). Brightness and contrast were applied equally across the entire image to both control and experimental groups using Photoshop.

Macrophages were outlined in F4/80-stained sections of liver and area was quantified using ImageJ by an investigator blinded to genotype and treatment.

Confocal imaging of tissue: One-week post intraventricular injection, vehicle or 5A-SM-treated mice were perfused with saline and tissues were placed in 4% PFA overnight. The liver and right hemisphere of the brain were embedded in OCT, frozen, and cut into 10-μm-thick sections. Sections were permeabilized (0.1% triton/10% NGS/1% BSA in PBS) for 30 min and placed into blocking buffer (10% NGS/1% BSA in PBS) for 60 min. Sections were placed in primary antibody overnight at 4 °C, washed three times in PBS for 5 min, then incubated in secondary antibody for 1 h at room temperature. Sections were stained with filipin and imaged on a Nikon A-1 confocal microscope. Purkinje neuron soma were defined using a calbindin-DK28 antibody, and filipin was quantified using ImageJ.

Stimulated Raman scattering (SRS) microscopy: Cell monolayers were imaged at 2845 cm^−1^ Raman shift wavenumber to generate a greyscale image channel. Images acquired at the 2845 cm^−1^ are chemically selective for lipids, stimulating vibrational resonance of the CH_2_ symmetric stretching mode [28]. Individual fields of view (FOVs) for lipid quantification were generated and quantified using a two-layer automated thresholding method to avoid selection bias. Over a full 2 mm × 2 mm SRS image, a 250 pixel × 250 pixel sliding window with 100-pixel step size was used to detect FOVs with greater than 90% cellular confluence. Mean background pixel intensity values for each image were used to set the FOV threshold for background (i.e., media) and foreground (i.e., cells). Only FOVs with a foreground/background ratio greater than 90% were included for lipid quantification. After selection of FOVs, a second thresholding procedure was used to segment intracellular lipid droplets, which have high 2845 cm^−1^ SRS signal compared to the remainder of the intracellular contents. For each FOV, a ratio between the area of intracellular lipid to total intracellular space was calculated and normalized to the number of cells within each image.

### Amplex Red

The Amplex® Red Cholesterol Assay Kit A12216 (Invitrogen) was used to quantify total free cholesterol following the manufacturer’s instructions.

### Cell death

Cell viability was assessed using Promega CellTiter 96 Aqueous One Solution cell proliferation colometric assay (G3580). Briefly, Niemann–Pick C cells were cultured in 96-well plates at 10,000 cells per well for 24 h, washed 3× with PBS, and treated as indicated with compounds diluted in media for 24 h. Cells were washed 3× with PBS and re-suspended in media supplemented with Promega CellTiter 96 reagent (20 μl reagent per 100 μl of media). After 45-min incubation at 37 °C, absorbance was read at 490 nm using a microplate reader. Each treatment was performed in triplicate, and the average absorbance reading of non-treated (Veh) cells was set to 100%. The percent viability was determined by dividing the average absorbance of treated over non-treated cells and multiplying by 100.

### Sphingomyelin loading

C6-NBD sphingomyelin was dissolved in 100% ethanol to make a 10 mM stock solution. Cells were treated with 40 μM C6-NBD sphingomyelin in cell culture media overnight. The following day (*t* = 0), wells were briefly washed 2× with PBS and fresh media without C6-NBD sphingomyelin was added. At *t* = 0 and *t* = 24 h, cells were treated with fresh media containing vehicle (saline) or 5A-SM.

### Radioactive cholesterol efflux assay

#### Preparation of [^3^H] cholesteryl linoleate-loaded acLDL

Cholesteryl [1,2,6,7-3H(N)] linoleate (60 Ci/mmol) was loaded into acetylated human LDL (acLDL) according to procedure adapted from Brown et al. [29]. Briefly, 30 μCi (0.5 nmol) cholesteryl [1,2,6,7-3H(N)] linoleate in toluene was evaporated to dryness under a stream of nitrogen gas. Then, a thin film of cholesteryl [1,2,6,7-3H(N)] linoleate was dissolved in 10 μl DMSO followed by the addition of 100 μl acLDL (5 mg of protein/ml). The mixture was incubated for 2 h at 37 °C with gentle shaking to incorporate cholesteryl [1,2,6,7-3H(N)] linoleate into acLDL and then dialyzed at 4 °C against 20 mM Tris/HCl, 0.3 mM EDTA, 0.15 M NaCl, pH 7.4 using 3.5K MWCO slide-A-Lyzer mini device (ThermoFisher 88,400). Cholesteryl [1,2,6,7-3H(N)] linoleate-acLDL mixture routinely contained 90–95% of starting radioactivity as determined by scintillation counting before and after dialysis.

#### Cholesterol efflux assay

Niemann–Pick C fibroblast cells were grown in culture media until confluency. On day 1, 75,000 cells were plated in 24-well plates and grown for 24 h in 0.5-ml culture media. On day 2, cells were washed with PBS, pH 7.4, 1× at room temperature and grown overnight in media containing lipoprotein-deficient serum (10% v/v) in DMEM to upregulate LDL receptors. On day 3, cells were washed with PBS, pH 7.4, 2×, and labeled with cholesteryl [1,2,6,7-3H(N)] linoleate-acLDL for 24 h in DMEM (no phenol red)/BSA (1 mg/ml)/P-S media (0.5 ml) containing 1 μCi of [^3^H] cholesteryl linoleate per 1-ml media. On day 4, labeled cells were washed with PBS, pH 7.4, 3×, to remove cholesteryl [1,2,6,7-3H(N)] linoleate not taken up by cells. Radioactive cholesterol was effluxed from cells for 24 h using vehicle (media), 5A peptide (0.75 mg/ml), 5A-SM HDL, 5A-DMPC, 5A-POPC (0.75 mg/ml), or cyclodextrin (1 mM) diluted in DMEM/BSA/P-S. On day 5, media from each well was transferred into separate Eppendorf tubes and centrifuged at 3000 rpm for 10 min to remove any detached cells. The remaining cells on the plate were lysed with 0.1% SDS/0.1 M NaOH solution for 2 h at room temperature. Radioactive counts of media and cell fractions were measured separately using a Perkin Elmer liquid scintillation counter. Percent cholesterol effluxed from cells was calculated by dividing media counts by the total sum of media and cell counts and then multiplying by 100%. Non-specific cholesterol efflux by vehicle was subtracted from all data.

### In vivo cholesterol mobilization

Total serum cholesterol concentrations from 7-week-old Niemann–Pick C mice pre- and 2 h post-treatment with 100 mg/kg 5A-SM i.p. were analyzed enzymatically by a colorimetric cholesterol oxidase assay (Wako Chemicals, Richmond, VA) using microplate reader.

### Distribution of mobilized cholesterol in lipoproteins

Serum samples from Niemann–Pick C mice collected at baseline and 2 h post-treatment with 100 mg/kg 5A-SM i.p. were analyzed to assess the cholesterol distribution between VLDL, LDL, and HDL lipoprotein fractions. Separation of lipoproteins from serum was performed on a Waters HPLC system equipped with a Superose 6, 10/300 GL column (GE Healthcare, Piscataway, NJ) and a fraction collector. Serum samples were injected onto the HPLC and eluted with saline solution pH 7.4 at 1 ml/min. Eluent fractions containing different lipoproteins were post-column reacted in the HPLC with an enzymatic solution for total cholesterol detection [30].

### Radioactive sphingomyelin efflux assay

Cells (40,000 cells/well) were cultured for 24 h in 24-well plates and then incubated with 1 μCi (80 Ci/mmol) sphingomyelin [choline methyl-3H] per 1-ml media. After 24 h, cells were washed with PBS, pH 7.4, 3×, followed by the treatment with vehicle or 0.75 mg/ml 5A-SM in culture media. Radioactivity in media and cells was counted using a PerkinElmer scintillation counter. Percent sphingomyelin effluxed from cells was calculated by dividing media counts by the total sum of media and cell counts and then multiplying this number by 100%. Non-specific sphingomyelin efflux by vehicle was subtracted from all data.

### Serum analysis

Whole blood was collected and allowed to clot for 5 min in BD microtainer® SST gold cap tubes (365967). Tubes were centrifuged for 5 min at 3000×*g* to remove the clot. Liver enzymes were blindly analyzed by the University of Michigan In-Vivo Animal Core.

### Statistics

Significance (*p* < 0.05) was determined by Graphpad Prism 7.0. Figure legends indicate when unpaired Student’s two-tailed *t* test, one-way or two-way ANOVA with Tukey, or Bonferroni post hoc analysis were used. All error bars are s.e.m. Graphpad outlier analysis was used to remove one outlier per group for Purkinje neuron filipin quantification.

## Results

### Design and synthesis of sHDL nanoparticles

ApoA1 enwraps lipids into 10–12-nm nanodiscs to form endogenous HDL [31]. These particles contain a heterogeneous mixture of saturated and unsaturated phospholipids, with each lipid having distinct cholesterol binding properties [32]. Relative to full-length ApoA1 protein, use of synthetic ApoA1 mimetic peptide is beneficial due to the ease of manufacturing, enhanced quality control, and lower cost [12]. We utilized the ApoA1 mimetic peptide 5A, which is designed to maximize cholesterol efflux by ABCA1 [22]. Using phospholipids with differing affinities for cholesterol [33], we developed a panel of sHDLs containing various 5A:lipid formulations, including sphingomyelin (SM), a saturated phospholipid (DMPC, 1,2-dimyristoyl-sn-glycero-3-phosphocholine), or an unsaturated phospholipid (POPC, 1-palmitoyl-2-oleoyl-glycero-3-phosphocholine).

**Fig. 1:**
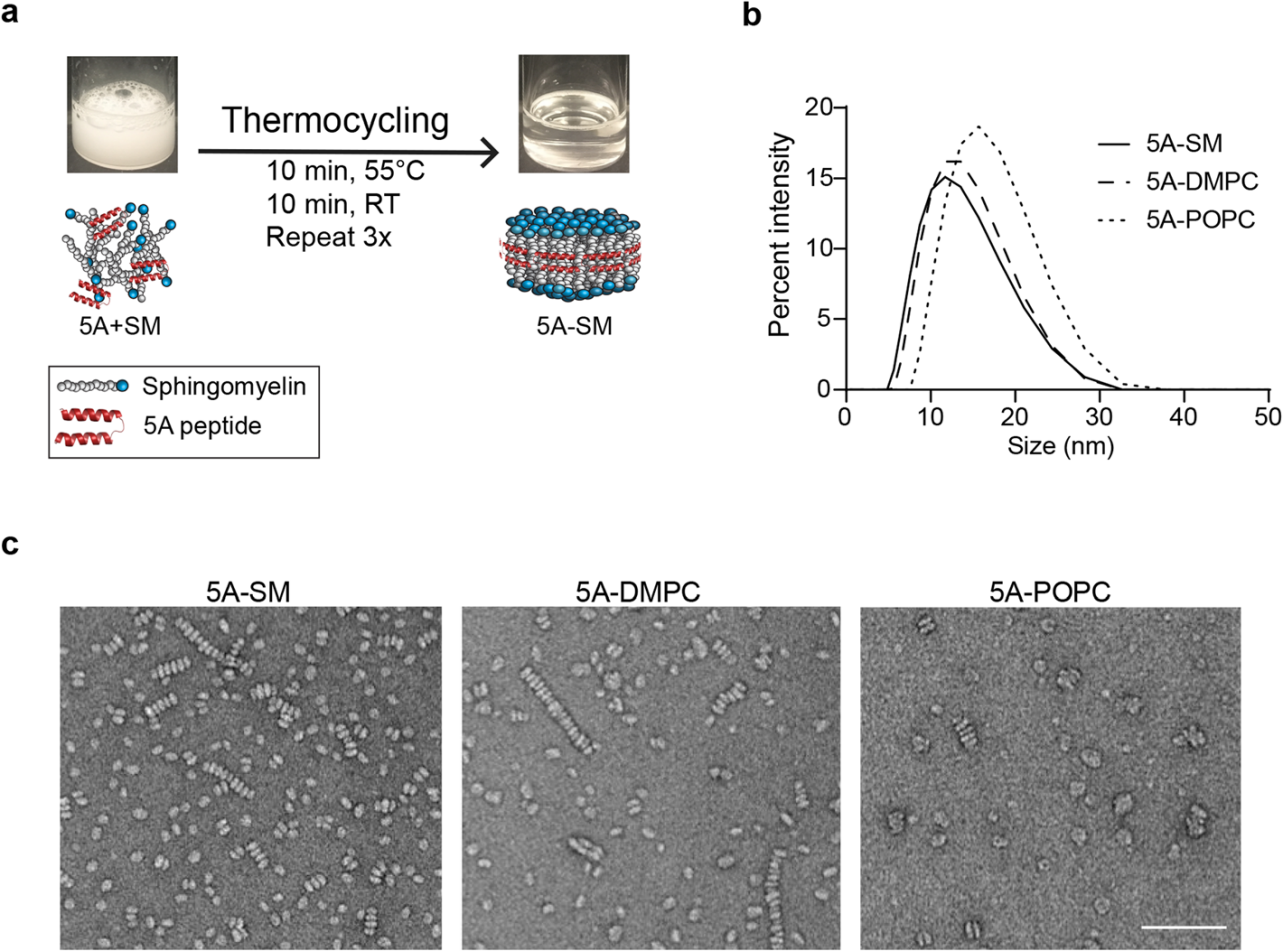
Synthesis and characterization of sHDL nanoparticles. **a** A lyophilized mixture of 5A peptide and sphingomyelin (SM) was hydrated in PBS and thermocycled to assemble sHDL particles. **b**, **c** Particle size distribution was analyzed by **b** dynamic light scattering for 1 mg/mL of 5A-SM, 5A-DMPC, or 5A-POPC sHDL or **c** transmission electron microscopy of 5A-SM, 5A-DMPC, and 5A-POPC. Scale bar = 100 nm

All sHDLs (5A-SM, 5A-POPC, and 5A-DMPC at 1:1.5 wt/wt ratio) were prepared by co-lyophilization and thermocycling (Fig. 1a) [14]. The lyophilization and thermocycling processes are highly efficient, and we detected little difference in the expected and actual ratios of peptide to lipid (Additional file 1: Table S1). sHDL particles had an average diameter of 10–12 nm (5A-SM and 5A-DMPC) as determined by dynamic light scattering (Fig. 1b). Hydrophobic uncomplexed lipids spontaneously form ≥ 100 nm liposomes. Large liposomes were not detected by dynamic light scattering (data not shown) indicating a highly efficient incorporation of lipids into sHDL. sHDLs also exhibited the expected size and disc-like morphology by transmission electron microscopy (Fig. 1c). sHDL “stacking” by transmission electron microscopy is likely an artifact of sample preparation since dynamic light scattering revealed monomeric sHDL in solution.

### sHDLs rescue cholesterol storage in Niemann–Pick type C fibroblasts

**Fig. 2:**
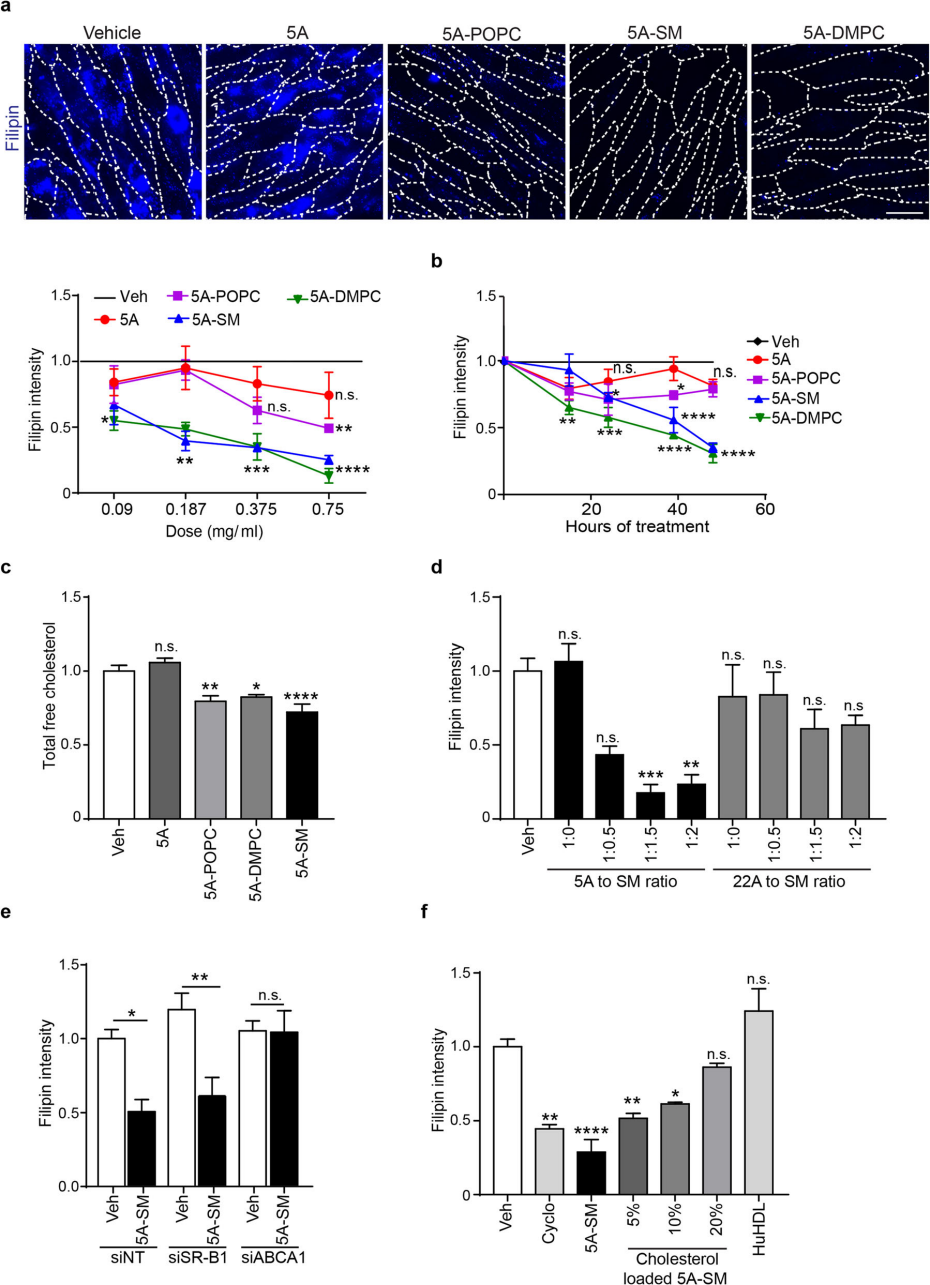
sHDLs require ABCA1 to remove accumulated cholesterol from Niemann–Pick C fibroblasts. **a**–**f** Primary fibroblasts homozygous for NPC1 I1061T were treated with various sHDL formulations. **a**, **b** Accumulation of unesterified cholesterol was visualized by filipin staining (**a**) following 48-h treatment with increasing doses (representative images of 0.75 mg/ml) of vehicle (Veh), 5A peptide, 5A-POPC, 5A-SM, and 5A-DMPC (quantified below) or (**b**) with 0.75 mg/ml sHDL at various time points. **c** Effects of 48-h treatment with sHDL (0.75 mg/ml), 5A peptide, or vehicle (Veh) on total cellular cholesterol were measured using the Amplex Red assay. **d** The ratio of 5A or 22A peptide to sphingomyelin (SM) was altered during synthesis and the effect of peptide: SM ratio on cholesterol removal was determined by filipin staining (48-h treatment). **e** Cells were treated for two consecutive days with the following siRNAs: non-targeting (NT), ABCA1, or SR-B1, and concurrently treated with vehicle (Veh) or 5A-SM. Cholesterol storage was determined by filipin staining. **f** Cells were treated with cyclodextrin (Cyclo), 5A-SM, or 5A-SM preloaded with increasing amount of cholesterol content (5–20% total lipid weight), or human HDL (HuHDL). Cholesterol storage was assessed by filipin staining 48 h after treatment. Data are mean ± s.e.m. from (**a**, **b**, **e**) three, (**c**) five, (**d**) 5–8, or (**f**) 4–6 independent experiments. n.s., not significant, *p ≤ .05, **p ≤ .01, ***p ≤ .001, ****p ≤ .0001 by **a**, **b** two-way ANOVA with Bonferroni post hoc test (*F*, df = (**a**) 33.53, df = 4; (**b**) 32.88, 4), **c**–**f** one-way ANOVA with Tukey post hoc test (*F*, df = (**c**) 13.98, 4; (**d**) 6.96, 8; (**e**) 22.5, 6; (**f**) 6.94, 5). **a** Dash lines indicate plasma membrane, scale bar = 20 μm

To assess the activity of sHDL on Niemann–Pick type C patient cells, we used the fluorescent dye filipin to label accumulated unesterified cholesterol. We analyzed filipin staining intensity in Niemann–Pick C fibroblasts following treatment with vehicle, 5A peptide alone, or sHDLs composed of 5A-POPC, 5A-SM, or 5A-DMPC. Treatment with 5A peptide alone did not significantly alter filipin intensity over 48 h (Fig. 2a, b). In contrast, both 5A-SM and 5A-DMPC significantly rescued stored cholesterol in a dose- and time-dependent manner in three independent lines of Niemann–Pick C primary fibroblasts (Fig. 2a, b, Additional file 1: Figure S1a). sHDL composed of 5A-POPC yielded a more modest and less consistent rescue, demonstrating that lipid composition impacts biological activity. The beneficial effects of sHDL treatment were confirmed using an amplex red assay to measure total cellular cholesterol (Fig. 2c). Assessment of cell viability following treatment showed no significant changes, except for mild toxicity from 5A-DMPC (Additional file 1: Figure S1b). Taken together, these studies demonstrate sHDL activity and tolerability, and given the investigational new drug status of 5A-SM, prompted further analysis of SM-containing sHDLs.

Although the lipid content of sHDL is a strong determinant of cholesterol efflux, the composition of the ApoA1 mimetic peptide is also important for sHDL function [17]. In order to investigate the impact of the ApoA1 mimetic peptide on sHDL’s ability to reduce cellular cholesterol storage, nanoparticles were prepared with another peptide, 22A [34, 35]. Both 5A and 22A peptides have no sequence homology with endogenous ApoA1 and were optimized differently: 5A peptide was selected to maximally efflux cholesterol by ABCA1, while 22A peptide was selected to maximize cholesterol esterification within sHDLs in plasma [16, 22, 35].

Raising the lipid to peptide ratio increases sHDL’s size and its capacity to accept cholesterol [15]. It is generally believed that larger sHDL particles efflux cholesterol through scavenger receptor B-1 (SR-B1) [36]. To examine the effect of peptide sequence and peptide to lipid ratio on cholesterol removal, we generated a panel of sHDLs containing 5A or 22A and various peptide to SM ratios. Filipin analysis revealed significant cholesterol-reducing activity of sHDLs containing 5A-SM but not 22A-SM. Moreover, we found that a 1:1.5 wt/wt ratio of 5A:SM was optimal at reducing filipin intensity (Fig. 2d).

To determine which cholesterol transporter is primarily responsible for 5A-SM mediated removal of unesterified cholesterol from Niemann–Pick C cells, we performed expression analysis of NPC patient fibroblasts. In contrast to ABCG1, expression of ABCA1 and SR-B1 were readily detected by qPCR (Additional file 1: Figure S1c). Next, we treated primary fibroblasts with non-targeting siRNAs (siNT), or siRNAs targeting SR-B1 (siSR-B1) or ABCA1 (siABCA1). 5A-SM efficiently rescued cholesterol storage after treatment with either siNT or siSR-B1, but not after treatment with siABCA1 (Fig. 2e). As siRNAs significantly reduced expression of target genes (Additional file 1: Figure S1d), this analysis confirmed that 5A-SM requires ABCA1 to function. Notably, increasing cholesterol content of 5A-SM reduced its effect on clearance of unesterified cholesterol (Fig. 2f). Consistent with this observation, incubation with a heterogeneous pool of human plasma HDL (HuHDL) containing nascent (cholesterol poor) and mature (cholesterol loaded) HDL failed to reduce cellular cholesterol levels (Fig. 2f). These data contrast the observation that the cholesterol reducing agent 2-hydroxypropyl-beta cyclodextrin (cyclodextrin) preloaded with cholesterol remains effective at reducing unesterified cholesterol in Niemann–Pick C cells [37–39], and raise that possibility that sHDL and cyclodextrin have distinct mechanisms of action.

### 5A-SM induces the expression of cholesterol regulatory genes

**Fig. 3:**
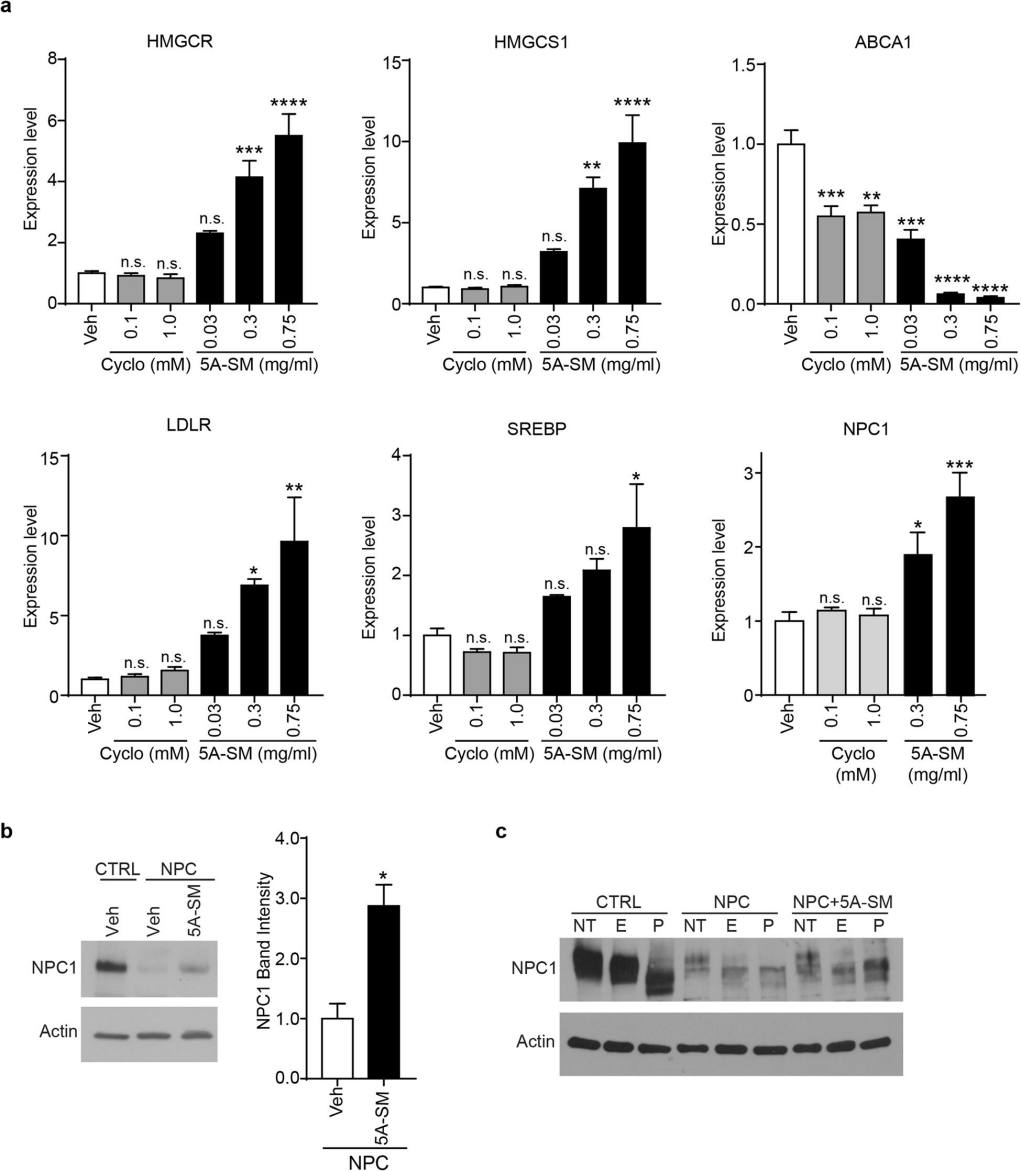
sHDLs modulate cholesterol regulatory genes. **a** Primary fibroblasts homozygous for NPC1 I1061T were treated with vehicle (Veh), cyclodextrin (cyclo), or 5A-SM at the indicated concentrations for 48 h. HMGCR, HMGCS1, ABCA1, LDLR, SREBP, or NPC1 mRNA expression was analyzed by qPCR. **b**, **c** NPC1 protein in control (CTRL) and NPC1 I1061T (NPC) fibroblasts was analyzed by **b** western blot (quantified at right) or **c** digested with endoglycosidase H (E), PNGase F (P), or not treated (NT) and analyzed by western blot. Data are mean ± s.e.m. from three independent experiments. n.s., not significant, *p ≤ .05, **p ≤ .01, ***p ≤ .001, ****p ≤ .0001 relative to Veh by **a** one-way ANOVA with Tukey post hoc test (*F*, df = (HMGCR) 27.43, df = 5; (HMGCS1) 24.75, 5; (ABCA1) 43.54, 5; (LDLR) 9.4, 5; (SREBP) 7.0, 5; (NPC1) 12.41, 4. **b** Student’s *t* test *t* = 3.83, df = 2

To more fully define the biological responses triggered by sHDLs, we treated Niemann–Pick C fibroblasts with increasing doses of cyclodextrin or 5A-SM and analyzed the expression of cholesterol regulatory genes. A 48-h treatment with cyclodextrin did not alter the expression of *HMGCR, HMGCS1,* or *LDLR* (Fig. 3a). This is consistent with previous research showing cyclodextrin causes a transient decrease then restoration in cholesterol biosynthetic genes by 48 h [37, 38, 40]. In contrast, 48 h of treatment with 5A-SM or 5A-DMPC caused a dose-dependent increase of cholesterol biosynthetic (*HMGCR, HMGCS1, SREBP*) and uptake genes (*LDLR*) in three independent lines of patient fibroblasts (Fig. 3a, Additional file 1: Figure S2a, b). Additionally, expression of the cholesterol export gene *ABCA1* was significantly decreased (Fig. 3a, Additional file 1: Figure S2a, b). Together, these data suggest that sHDLs efficiently extract cholesterol from target cells and act at different time scales than cyclodextrin.

Treatment with 5A-SM also significantly increased the expression of *NPC1* mRNA and protein (Fig. 3a, b, Additional file 1: Figure S2c, d). This is notable since multiple groups have shown that a subset of NPC1 missense mutants are functional if they escape ER degradation and traffic to LE/Lys [41, 42]. To determine whether the induction of NPC1 contributed to rescue of lipid storage, we took advantage of the fact that the NPC1 protein is heavily glycosylated. These glycans are modified as the protein traffics through the medial Golgi, rendering them resistant to cleavage by endoglycosidase H (EndoH) but maintaining sensitivity to PNGaseF. As expected, wild-type (WT) NPC1 protein expressed in control fibroblasts was resistant to EndoH cleavage, while mutant NPC1 protein from patient fibroblasts was sensitive to EndoH (Fig. 3c). Treatment with 5A-SM did not alter the sensitivity of mutant NPC1 to digestion by EndoH, indicating that the accumulated protein did not traffic to LE/Lys (Fig. 3c, Additional file 1: Figure S2e). We conclude that 5A-SM removes cholesterol from patient fibroblasts without correcting mutant NPC1 protein trafficking or function.

### 5A-SM enters cells through macropinocytosis and promotes cholesterol efflux

**Fig. 4:**
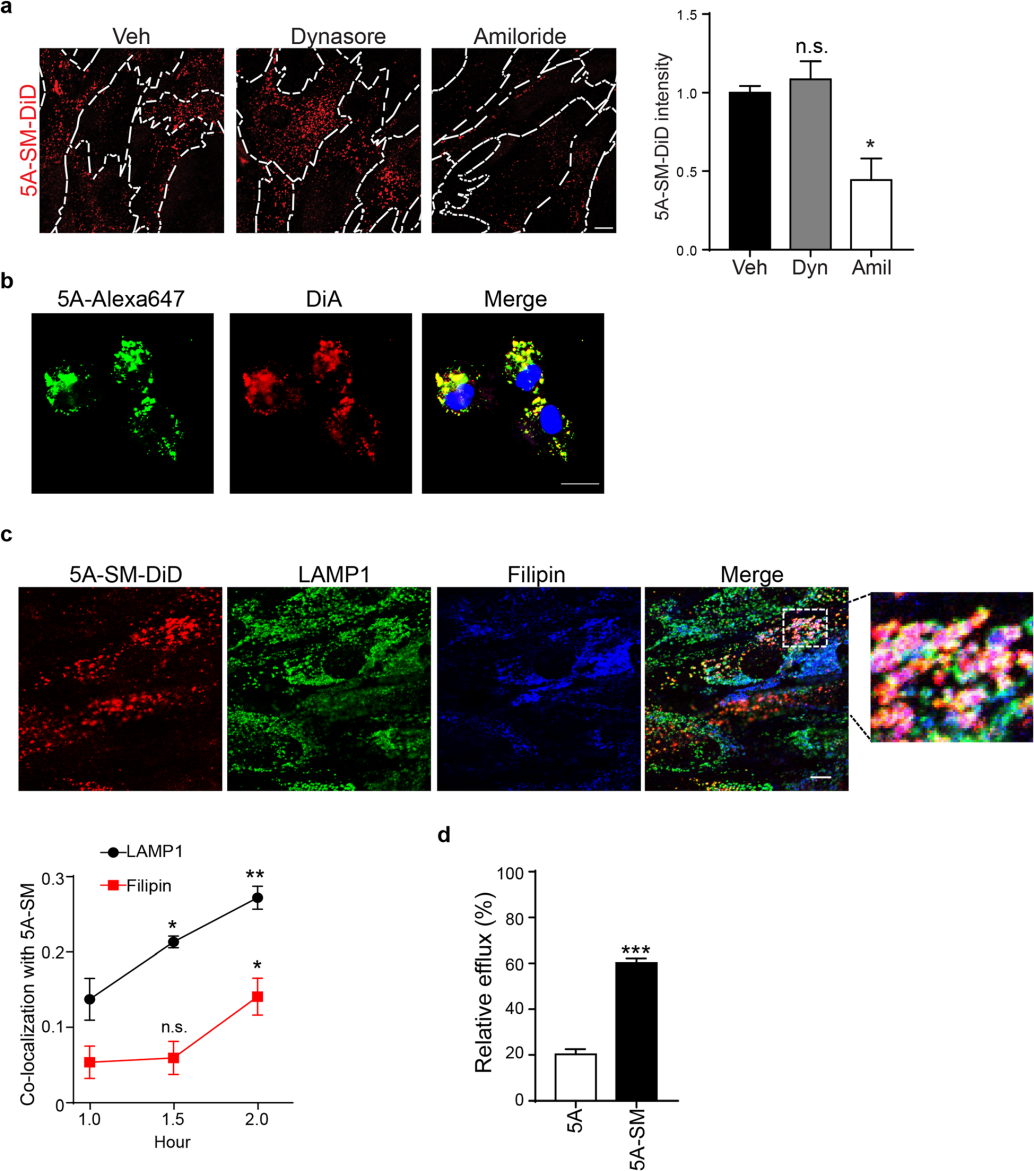
5A-SM is endocytosed and increases cholesterol efflux. **a**–**d** NPC1 I1061T fibroblasts were treated with the indicated sHDL for **a**–**c** 2 or **d** 24 h. **a** Cells were pre-treated with dynasore (80 μM), amiloride (1 mM), or vehicle (Veh) for 30 min and then incubated with fresh media containing 5A-SM-DiD plus dynasore, amiloride, or vehicle for 2 h. Plasma membranes are outlined with dashed lines. 5A-SM-DiD (red) intensity is quantified at the right. **b** Cells were treated with sHDL composed of 5A-Alexa647 (green) and DiA (red) incorporated into the SM fraction. Following 2-h incubation, cells were labeled with NucStain (blue) and imaged by confocal microscopy. Pearson co-localization coefficient = 0.75 ± 0.01. **c** Cells were incubated with 5A-SM-DiD (red) for 1, 1.5, and 2 h, fixed, stained for LAMP1 (green) and filipin (blue), and imaged by confocal microscopy. Representative images from 2 h post-treatment. Pearson co-localization coefficient quantified below. **d** Cells were pre-treated for 24 h with acetylated LDL containing [3H] cholesteryl linoleate to specifically deliver cargo to the lysosomal compartment. Following 24-h equilibration, cells were treated for 24 h with 0.75 mg/ml 5A peptide or 5A-SM. Radioactivity in media and cell fractions was determined by liquid scintillation counting, and values were normalized to vehicle treated group. Data are mean ± s.e.m. from three independent experiments. n.s., not significant, *p ≤ .05, **p ≤ .01, ****p ≤ .0001 by **a** one-way ANOVA with Tukey post hoc test relative to Veh or 5A (*F* = 10.74, df = 2); **c** two-way ANOVA with Bonferroni post hoc test (*F*, df = 23.63, 2). **d** Student’s *t* test *t* = 13.09, df = 4. Scale bar = **a** 12 μm, **b** 20 μm, **c** 10 μm

Cholesterol is loaded into nascent HDL particles when ApoA1 interacts with receptors such as ABCA1 at the plasma membrane. However, a prior report indicates that ApoA1 and ABCA1 may be endocytosed as a complex [43]. Studies have suggested that ApoA1/ABCA1 endocytosis is required for removal of accumulated LDL-derived cholesterol from LE/Lys [43, 44]. To determine if 5A-SM enters cells through endocytosis, we treated Niemann–Pick C patient fibroblasts with 5A-SM sHDLs containing the fluorescent lipophilic dye DiD (5A-SM-DiD). Confocal imaging revealed little 5A-SM-DiD signal on the plasma membrane, yet readily identified fluorescent signal in the cytoplasm, indicating uptake of the 5A-SM-DiD nanoparticles (Fig. 4a). To define the mechanism of uptake, cells were pretreated with the macropinocytosis inhibitor amiloride [45] or the clathrin and caveolar inhibitor dynasore, then loaded with 5A-SM-DiD. Dynasore had little effect on 5A-SM-DiD uptake, while amiloride significantly reduced 5A-SM-DiD signal intensity, indicating that macropinocytosis is a major route of 5A-SM-DiD endocytosis (Fig. 4a). Notably, the lipophilic tag DiD was not covalently conjugated to 5A-SM; therefore, the punctate cytoplasmic pattern of fluorescence could represent DiD dissociated from the nanoparticle. To rule out this possibility, we synthesized sHDLs containing 5A peptide covalently conjugated to Alexa647 (5A-Alexa647) and incorporated the lipophilic dye DiA into these particles (5A:Alexa647-SM:DiA). After 2-h incubation, the 5A and DiA signals strongly co-localized (Fig. 4b) indicating that the internalized 5A-SM sHDL particles remained intact inside the cell.

Unesterified cholesterol that accumulates within Niemann–Pick C cells resides in LAMP1-positive LE/Lys [39]. To determine if 5A-SM traffics to this compartment, we conducted a time course and analyzed co-localization of 5A-SM-DiD with LAMP1 and filipin. We observed that a fraction of 5A-SM-DiD co-localized with LAMP1 and filipin-positive lipid storage vesicles over a period of 2 h (Fig. 4c). At this time point, 5A-SM-DiD did not co-localize with the recycling endosome/early endosome marker EEA1 (Additional file 1: Figure S3), possibly because it had already traveled past this compartment. Whether lysosomal/filipin-positive 5A-SM-DiD compartments represent the primary site of sHDL action requires further investigation, and it remains possible that sHDLs act at other intracellular sites. In either case, we sought to confirm that sHDL uptake was accompanied by efflux of stored cholesterol. To accomplish this, patient fibroblasts were loaded and then equilibrated with [3H] cholesteryl linoleate bound to LDL. We treated cells with 5A or 5A-SM for 24 h and then measured intracellular and extracellular [3H]cholesterol. Incubation with 5A peptide alone resulted in ~ 20% efflux of radiolabeled LDL-derived cholesterol to medium (Fig. 4d). Notably, pre-formed 5A-SM sHDL particles were much more effective at effluxing LDL [3H] cholesterol than 5A alone, resulting in release of ~ 60% of labeled cholesterol into the media. In contrast, 24-h treatment with 1 mM cyclodextrin resulted in a modest 6.2% ± 2.7 efflux of LDL-derived cholesterol at this early time point. This is in line with previous data showing that cyclodextrin extracts cholesterol from the plasma membrane and can mobilize it from intracellular stores [39, 40, 46].

### 5A-SM mobilizes cholesterol and ameliorates phenotypes in Niemann–Pick C mice

**Fig. 5:**
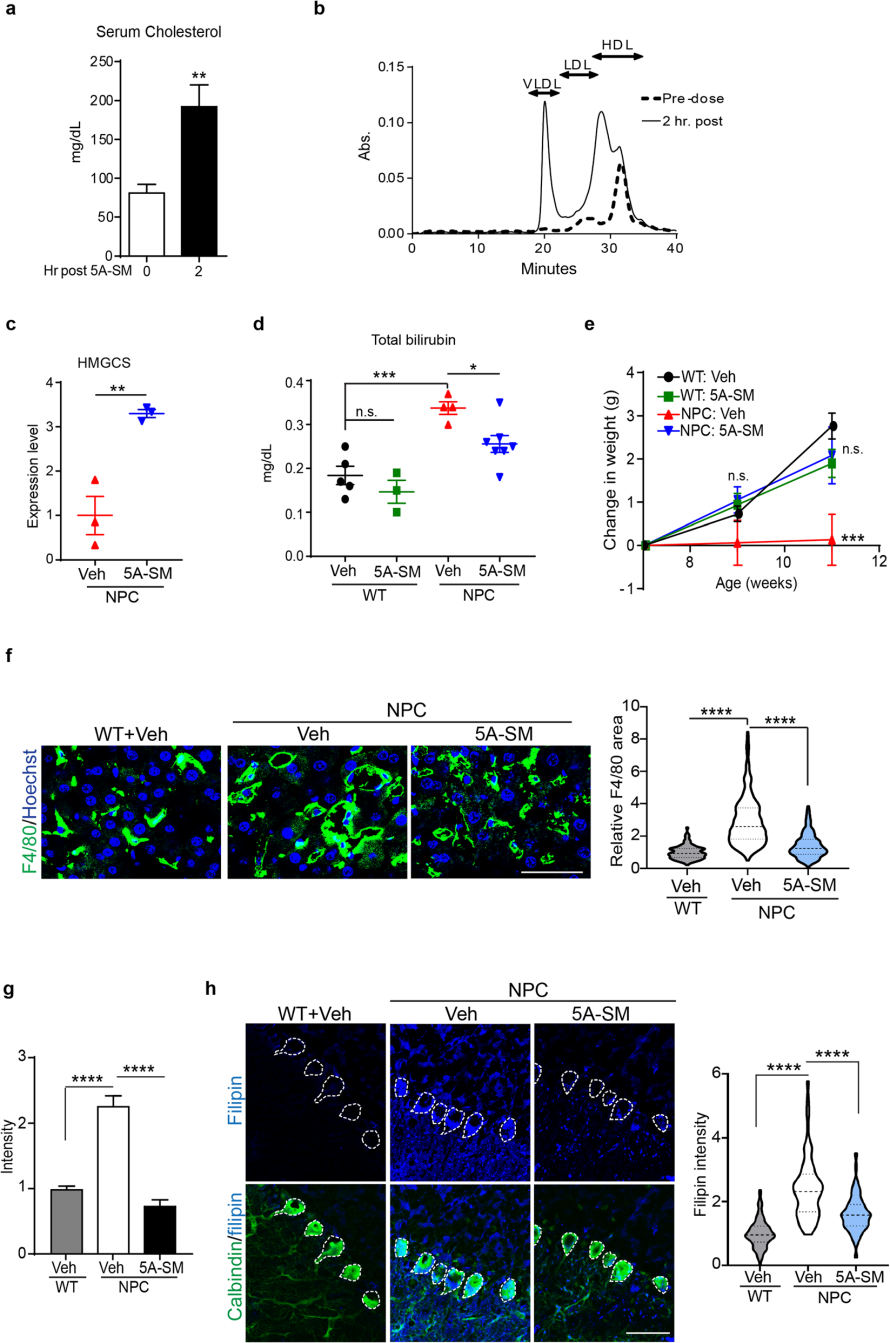
5A-SM mobilizes cholesterol in vivo and ameliorates disease phenotypes*.* **a** Serum cholesterol from 7-week-old Npc1 I1061T homozygous mice pre- and 2 h post-treatment with 100 mg/kg 5A-SM, i.p. **b** Pre- (dashed line) and 2 h post-treatment (solid line) serum was fractionated by HPLC, and cholesterol was quantified by cholesterol oxidase assay. VLDL, LDL, and HDL fractions are indicated by arrows. **c**, **d** Seven-week-old wild-type (WT) and Npc1 I1061T homozygous (NPC) mice were injected i.p. with vehicle (Veh) or 100 mg/kg 5A-SM. 48 h later, **c** liver HMGCS transcript levels and **d** total serum bilirubin were analyzed. **e** WT and Npc1 I1061T mice were injected i.p. with vehicle (Veh) or 100 mg/kg 5A-SM three times per week from 7 to 11 weeks of age. The change in weight of each mouse from week 7 (*t* = 0) to week 11 (*t* = 4) was quantified. **f** Seven-week-old WT and NPC mice were injected with Veh or 100 mg/kg 5A-SM three times per week for 2 weeks. At 9 weeks of age livers were stained for macrophages using F4/80 (green) and DNA Hoechst (blue). Macrophage area is quantified at right. Scale bar = 50 μm. Violin plot shows median (dashed line), 25% and 75% (dotted lines), and probability density (thickness). **g** Brain slices from 8-week-old Npc1 I1061T mice were incubated with vehicle (Veh) or 5 mg/ml 5A-SM for 4 days, and filipin levels in Purkinje neuron soma were quantified (see also Additional file 1: Figure S4b). **h** Six- to 7-week-old WT and Npc1 I1061T mice received intraventricular injections with vehicle (Veh) or 5A-SM-DiD. N: WT = 4, NPC Veh = 5, NPC 5A-SM = 4 mice. One-week later, cholesterol levels in Purkinje neuron soma (green) were analyzed by filipin (blue) staining. Dashed lines indicate Purkinje neuron soma (also see Additional file 1: Figure S6a). Scale bar = 50 μm. Data quantified at right. Data are mean ± s.e.m. from **a**, **b**, **c** three; **d** genotype and treatment: number of mice, WT + Veh = 5, WT + 5A-SM = 3, NPC + Veh = 4, NPC + 5A-SM = 7; **e** genotype and treatment: number of mice at 9 weeks and 11 weeks, WT + Veh: 13 and 8, WT + 5A-SM: 9 and 8, NPC + Veh: 6, NPC + 5A-SM: 12 and 10 mice; **f** genotype and treatment: number of mice, cells, WT + Veh: 4, 301, NPC + Veh: 4, 514, NPC + 5A-SM: 3, 373 (**g**) WT = 93, NPC Veh = 143, NPC + 5A-SM = 116 cells. *p ≤ .05, **p ≤ .01, ***p ≤ .001, ****p ≤ .0001. **a**, **c** Student’s *t* test (*t* = (**a**) 6.375, (**c**) 5.23); **d**, **f**, **g**, **h** one-way ANOVA with Tukey post hoc test (*F*, df = (**d**) 13.28, 3; (**f**) 368.1, 2 (**g**) 38.89, 2; (**h**) 108.3, 2); **e** two-way ANOVA with Bonferroni post hoc test (*F*, df = 7.12, 2)

Based on the significant rescue of cholesterol storage observed in vitro, we sought to determine the extent to which administration of sHDLs benefit gene targeted mice homozygous for the *Npc1 I1061T* allele (Niemann–Pick C mice). These mice contain the most common Niemann–Pick C disease-causing mutation (I1061T). Starting at 7 weeks of age, these mice develop robust, progressive phenotypes including cholesterol accumulation, Purkinje neuron loss, motor impairment, and premature death by 13 weeks of age [24]. Niemann–Pick C mouse serum collected pre- and 2 h post-intraperitoneal (i.p.) injection of 5A-SM established that treatment significantly increased serum cholesterol content (Fig. 5a). High-performance liquid chromatography (HPLC) was used to identify cholesterol-containing fractions (VLDL, LDL, or HDL). Serum cholesterol was distributed in all lipoprotein fractions 2 h post 5A-SM injection, with LDL and VLDL particles containing the most cholesterol (Fig. 5b). This is a typical lipoprotein profile after sHDL administration, where immediate HDL-cholesterol elevation is followed by transient LDL/VLDL-cholesterol elevation while cholesterol is metabolized, returning to baseline 24 h post-treatment [14, 15, 19]. Consistent with data showing cholesterol mobilization into the serum, a single injection of 5A-SM significantly upregulated the cholesterol biosynthetic gene *HMGCS* in the liver (Fig. 5c), similar to our findings in patient fibroblasts (Fig. 3a). These data provide evidence of target engagement after in vivo administration of sHDL to Niemann–Pick C mice.

To determine whether Niemann–Pick C mice showed benefits from sHDL administration, we initially focused on peripheral disease manifestations that might be responsive to i.p. administration. Niemann–Pick C mice exhibit significantly elevated serum bilirubin levels, diminished body weight, and liver macrophage activation. We analyzed total bilirubin levels in 7-week-old WT and Niemann–Pick C mice 48 h after a single injection of vehicle or 5A-SM. sHDL administration rescued bilirubin levels in Niemann–Pick C mice without altering them in WT littermates (Fig. 5d). Niemann–Pick C mice also exhibit progressive body weight loss as they age [24]. To determine effects on this disease manifestation, 5A-SM or vehicle was injected i.p. three times per week from 7 to 11 weeks of age, and the change in weight was calculated for each mouse for the duration of the treatment trial. WT mice treated with either vehicle or 5A-SM gained ~ 2 g during the treatment period, whereas vehicle treated Niemann–Pick C mice failed to gain body weight. In contrast, Niemann–Pick C mice treated with 5A-SM exhibited a significant rescue of body weight, gaining as much weight as WT controls (Fig. 5e). Administration of 5A-SM also significantly reduced liver macrophage size in Niemann–Pick C mice (Fig. 5f). While i.p. administration of sHDL showed significant benefits for these peripheral phenotypes, it did not correct motor phenotypes (Additional file 1: Figure S4a), suggesting poor blood–brain barrier penetration.

The Niemann–Pick C mouse motor phenotypes are driven, in part, by loss of cholesterol-laden cerebellar Purkinje neurons [47, 48]. To determine whether sHDLs could rescue cholesterol storage in neurons, we treated cultured cerebellar slices from adult WT and Niemann–Pick C mice with vehicle or 5A-SM for 4 days. Slices were fixed and co-labeled for Purkinje neurons (calbindin) and cholesterol (filipin). Confocal imaging demonstrated that treatment of Niemann–Pick C brain slices significantly reduced cholesterol storage in Purkinje neurons (Fig. 5g and Additional file 1: Figure S4b). This finding demonstrates that sHDLs are active on CNS target cells if they gain access to the brain. To directly test CNS activity, we performed intraventricular injections in 6–7 week-old Niemann–Pick C mice with vehicle or 5A-SM containing the fluorescent dye DiD (5A-SM-DiD), a manipulation that was well tolerated. One week post-injection, 5A-SM-DiD signal localized to the cerebellum, brain stem, cortex, and hippocampus (Additional file 1: Figure S5). In the cerebellum, the fluorescent signal from DiD localized predominantly to astrocytes and, to a lesser extent, microglia at this time point (Additional file 1: Figure S6b, c). We calculated Purkinje neuron soma size as a potential indicator of in vivo toxicity of 5A-SM 1 week post injection. Suggesting little toxicity, Purkinje neuron soma size was unchanged with 5A-SM treatment (WT + Veh, 185 ± 44; NPC + Veh, 207 ± 57; and NPC + 5A-SM, 206 ± 61 pixels). One week post-injection, we observed significant reduction of cholesterol accumulation in Purkinje neurons in Niemann–Pick C mice treated with sHDL (Fig. 5h).

### 5A-SM reduces accumulated sphingomyelin in Niemann–Pick A fibroblasts

Both cholesterol and sphingomyelin utilize the ABCA1 transporter to efflux from cells into an HDL acceptor [49]. These two lipids physically interact and commonly traffic together [50]. This suggested that sHDL might be effective at rescuing aberrant storage of sphingomyelin as well as cholesterol. Sphingomyelin is normally metabolized by the lysosomal enzyme acid sphingomyelinase, and loss-of-function mutations in the encoding gene result in sphingomyelin accumulation, causing Niemann–Pick disease types A and B [51].

**Fig. 6:**
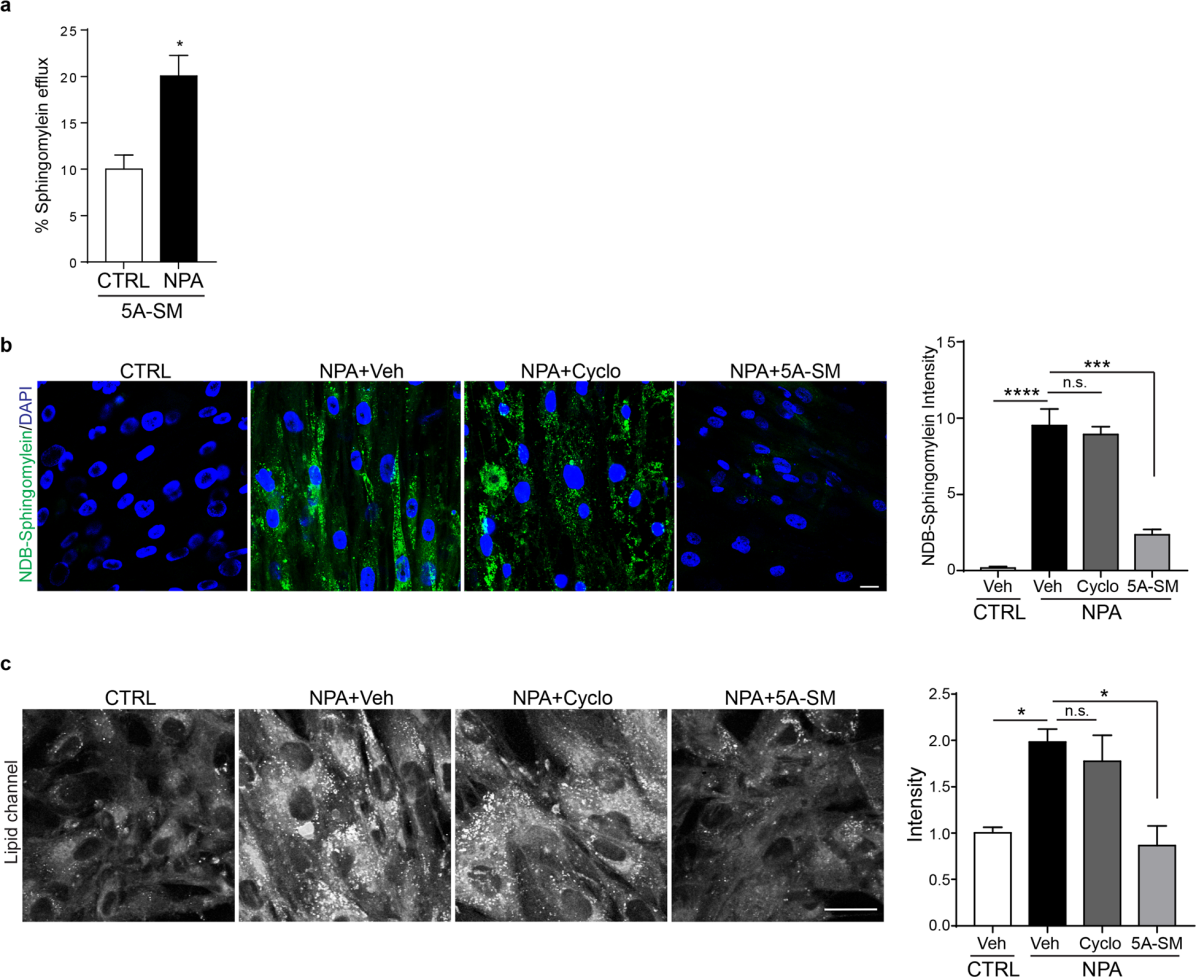
5A-SM removes sphingomyelin from Niemann–Pick type A fibroblasts. **a** Control (CTRL) and Niemann–Pick A (NPA) primary fibroblasts were incubated with [3H] sphingomyelin for 24 h, followed by treatment with 0.75 mg/ml 5A-SM or vehicle (Veh). Radioactivity in media and cell fractions was determined by liquid scintillation counting. **b** CTRL and NPA primary fibroblasts were loaded with NBD-sphingomyelin, then treated for 48 h with vehicle (Veh), cyclodextrin (Cyclo), or 0.75 mg/ml 5A-SM. NBD-sphingomyelin intensity quantified at right. Scale bar = 20 μm. **c** SRS microscopy was used to image total endogenous lipids in CTRL and NPA cells with the indicated treatments. Quantified at right. Scale bar = 20 μm. Data are mean ± s.e.m. from **a**, **b**, **c** three; *p ≤ .05, ***p ≤ .001, ****p ≤ .0001. **a** Student’s *t* test (*t* = 6.04); **b**, **c** one-way ANOVA with Tukey post hoc test (*F*, df = (**b**) 55.57, 3; (**c**) 8.285, 3)

To determine whether sHDL was capable of removing stored sphingomyelin from cells, Niemann–Pick A and control primary fibroblasts were loaded with [3H] sphingomyelin for 24 h and then treated with 5A-SM. After 24 h, 5A-SM promoted the efflux of twice as much [3H] sphingomyelin from Niemann–Pick A cells than control cells (Fig. 6a). To confirm this observation, Niemann–Pick A fibroblasts were loaded overnight with fluorescent NBD-sphingomyelin. Cells were then treated with vehicle, cyclodextrin, or 5A-SM for 48 h (Fig. 6b). As expected, control cells metabolized NBD-sphingomyelin and had little signal, while Niemann–Pick A cells showed marked cytoplasmic accumulation. Remarkably, 5A-SM significantly reduced NBD-sphingomyelin storage in Niemann–Pick A fibroblasts. In contrast, cyclodextrin treatment was ineffective, consistent with previous work in other sphingolipidoses [52].

The addition of exogenous sphingomyelin can alter membrane dynamics and impact downstream protein function [53, 54]. Therefore, we sought to determine effects of 5A-SM on endogenous lipids stored in Niemann–Pick A cells. To detect total lipids in a live, unfixed state, we utilized stimulated Raman scattering (SRS) microscopy. This SRS system generates virtual histology images that are useful for a variety of applications, including the clinical setting [55]. SRS utilizes an excitation and pump beam at the Raman wave number for CH2 bonds to rapidly image total lipids in biological samples. Since sphingomyelin contains extensive CH2 bonds, we developed SRS cell plating, imaging, and analysis procedures for Niemann–Pick A fibroblasts. SRS imaging demonstrated that Niemann–Pick A fibroblasts had double the lipid signal intensity compared to control cells (Fig. 6c). Treatment of Niemann–Pick A fibroblasts with 5A-SM rescued this lipid storage, whereas treatment with cyclodextrin did not. Collectively, these data show that 5A-SM is effective at reducing sphingomyelin storage in Niemann–Pick A cells and suggest that sHDL nanoparticles may be therapeutically beneficial for the family of Niemann–Pick diseases.

## Discussion

We describe an innovative approach to ameliorating lipid storage in the Niemann–Pick family of diseases by harnessing the activity of the body’s endogenous cholesterol scavenging particle, HDL. The sHDL particles characterized here potently remove stored cholesterol from Niemann–Pick C fibroblasts (Fig. 2) and neurons (Fig. 5f). The particles show evidence of cholesterol target engagement and rescue disease phenotypes when administered to Niemann–Pick C mice (Fig. 5). The 10–12 nm sHDL nanodiscs are generated at high purity by assembling peptide-lipid nanoparticles by a co-lyophilization and thermocycling process (Fig. 1). Notably, the degree of cholesterol removal was affected by altering the constituent ApoA1 mimetic peptide, lipid, and peptide to lipid ratio (Fig. 2), demonstrating that sHDLs provide a flexible platform that can be tuned to adjust therapeutic potency. Moreover, our observation that the sHDL that rescues cholesterol storage in type C disease also rescues sphingolipid storage in type A disease (Fig. 6) raises the possibility that alternative sHDL compositions may be beneficial for additional lipid storage disorders. The initial in vivo analyses presented here provide a proof of concept of activity for a single sHDL formulation, 5A-SM, at limited points. 5A-SM treatment of Niemann–Pick C mice induces cholesterol mobilization from the liver (increased HMGCS expression, Fig. 5c), increases serum cholesterol (Fig. 5a), and reduces liver inflammation (Fig. 5f). These data set the stage for additional analyses in Niemann–Pick animal models, including comparisons with other therapies currently administered to patients or in clinical trial. Future analyses are also needed to determine the extent to which optimized sHDL treatment regimens impact lysosomal cholesterol and sphingolipid storage in liver and normalize oxysterol biomarkers.

Prior studies have established that Niemann–Pick type C cells have normal ApoA1 receptor binding, endocytosis, and re-secretion [56, 57], yet Niemann–Pick patients have reduced serum HDL levels [56, 58–62] that likely worsen lipid storage. Previous reports have also demonstrated that Niemann–Pick type C cells are defective in loading cholesterol into ApoA1 [56, 57, 63]. Similarly, we noted that ApoA1 mimetic peptides are not sufficient for reducing Niemann–Pick C cholesterol storage (Fig. 2a, b). Our strategy bypassed the HDL formation deficiencies in disease by utilizing an investigational new drug, 5A-SM sHDL, that exhibits no significant cellular toxicity. The activity of alternative sHDL formulations will be the subject of future research. Additionally, the extent to which incorporation of ApoE rather than ApoA1 mimetic peptides and the addition of brain targeting peptides enhance therapeutic efficacy in the CNS remain to be defined.

While the rescue of cholesterol storage from mutant fibroblasts required expression of ABCA1 (Fig. 2e), fluorescently labeled sHDLs readily entered cells by macropinocytosis (Fig. 4a). The lipid and peptide constituents of the nanoparticles remained tightly associated within cells (Fig. 4b, c), with some trafficking to LAMP1 and filipin staining vesicles (Fig. 4c). Other intracellular sHDLs remained outside these lipid storage vesicles, and the precise location of cholesterol loading remains to be determined. In contrast to LDL, there is still incomplete understanding of HDL endocytosis and subcellular trafficking [64]. Interestingly, multiple intracellular pools of HDL have been described. After endocytosis, HDL can be re-secreted, sent to the lysosome, or trafficked to the Golgi before re-secretion [64–66]. The location of the subcellular pools of HDL is still being described and is likely cell type dependent. Although we were not able to define where all of the 5A-SM localized within cells, treatment with sHDLs did trigger cholesterol efflux (Fig. 4d), a finding that may reflect release of cholesterol-laden nanoparticles and/or enhanced lysosomal exocytosis.

The identification of therapeutic rescue of lipid storage in Niemann–Pick type A cells by sHDL was greatly facilitated by SRS microscopy (Fig. 6c). This technique was used to circumvent challenges associated with labeling endogenous sphingolipids and shortcomings of applying exogenous sphingolipids to study intracellular trafficking. We were not able to identify the accumulated lipid species by current technology and cannot exclude the possibility that sphingomyelin correction is a consequence of cholesterol removal. However, this seems unlikely as treatment with the cholesterol-removing agent cyclodextrin did not alter sphingomyelin accumulation or lipid content in Niemann–Pick type A cells (Fig. 6b, c). Notably, limited techniques allow live cell imaging of endogenous lipids. While SRS cannot currently delineate lipid subspecies, we anticipate that continued development of this technology will enable this process. Moreover, the robust rescue of lipid storage in Niemann–Pick type A cells, along with the amelioration of peripheral phenotypes in Niemann–Pick C mice following i.p. administration, is particularly encouraging in the context of Niemann–Pick type B. Niemann–Pick B is characterized by peripheral organ system phenotypes, but not CNS involvement. Even with the successful removal of sphingomyelin in Niemann–Pick A cells by 5A-SM, future research is needed to establish the optimal sHDL formulation for removing stored lipids in this disorder.

## Conclusion

Our study demonstrates a proof of concept that sHDL significantly reduce lipid storage in both Niemann–Pick C and A. Future work will assess the long-term effects of sHDL in NPC and NPA/NPB mouse models. Considering the safety of sHDL in clinical trials, correction of peripheral phenotypes would justify testing large animals and perhaps patients with these disorders. As such, our data suggest that 5A-SM or other sHDLs may be therapeutically beneficial for patients with Niemann–Pick diseases and possibly other lysosomal storage disorders.

## Supplementary information

**Additional file 1: Table S1.** Expected and actual peptide: lipid ratios. **Figure S1.** sHDLs rescue cholesterol storage in Niemann-Pick C patient fibroblasts. **Figure S2.** sHDLs induce the expression of cholesterol regulatory genes.** Figure S3.** 5A-SM-DiD does not co-localize with EEA1. **Figure S4.** Effects of 5A-SM treatment. **Figure S5.** 5A-SM distribution in the brain after ICV injection. **Figure S6.** Cellular distribution of 5A-SM after ICV injection.

## Data Availability

The data supporting the findings of this study are available from the corresponding author upon reasonable request. Python code for image tiling and lipid quantification can be found at github.com/toddhollon/lipid_quant_SRH.
