# Supplementary material for: Synthetic high-density lipoprotein nanoparticles for the treatment of Niemann–Pick diseases
Source: BMC Med. 2019 Nov 11;17:200. doi: 10.1186/s12916-019-1423-5 (PMC6849328; doi:10.1186/s12916-019-1423-5)

Additional file 1:

Synthetic high-density lipoprotein nanoparticles for the treatment of Niemann-Pick diseases

Schultz et. al.

**Methods**

*Phospholipid and Peptide Concentration in sHDL*: Phospholipid content in each formulation was determined using a FUJIFILM Wako Phospholipids C kit (cat# 997-01801) according to the manufacturer’s instructions. Peptide content was measured via UPLC (Waters Acquity UPLC) on a BEH C18 4.6 x 150mm column with a gradient from 15% A 85% B to 85% A 15% B (A= 0.1% TFA in ACN, B= 0.1% TFA in H20) over the course of 2 minutes and held for 0.5 minutes. Peptides were detected by UV absorbance at 215 and 280 nm.

*Balance beam*; training: 6 week old mice were acclimated inside a dark plastic hut containing bedding for five min. Mice were placed on a clear platform and encouraged to cross a 1 cm diameter round beam 3 X with one min rests between each trial.

Testing: The training protocol was repeated at 7, 9, 11, and 13 weeks of age. Time to cross from one platform to the other using the beam was recorded and averaged for each mouse. The maximum time to cross the beam was set at 20 sec.

| **sHDL Formulation** | **Peptide Concentration (mg/mL)** | **Phospholipid Concentration (mg/mL)** | **Expected Peptide:Lipid Ratio** | **Actual Peptide:Lipid Ratio** |
| --- | --- | --- | --- | --- |
| 5A-SM 1 | 11.96 | 6.12 | 1:0.5 | 1:0.51 |
| 5A-SM 2 | 10.13 | 8.91 | 1:1 | 1:1.14 |
| 5A-SM 3 | 10.84 | 15.26 | 1:1.5 | 1:1.41 |
| 5A-POPC | 9.33 | 13.5 | 1:1.5 | 1:1.45 |
| 5A-DMPC | 11.51 | 17.28 | 1:1.5 | 1:1.50 |

**Table Supplementary 1. Expected and actual peptide: lipid ratios.** Actual peptide and lipid composition were empirically determined following the “*Phospholipid and Peptide Concentration in sHDL”* protocol outlined in Additional file 1: Methods.

**
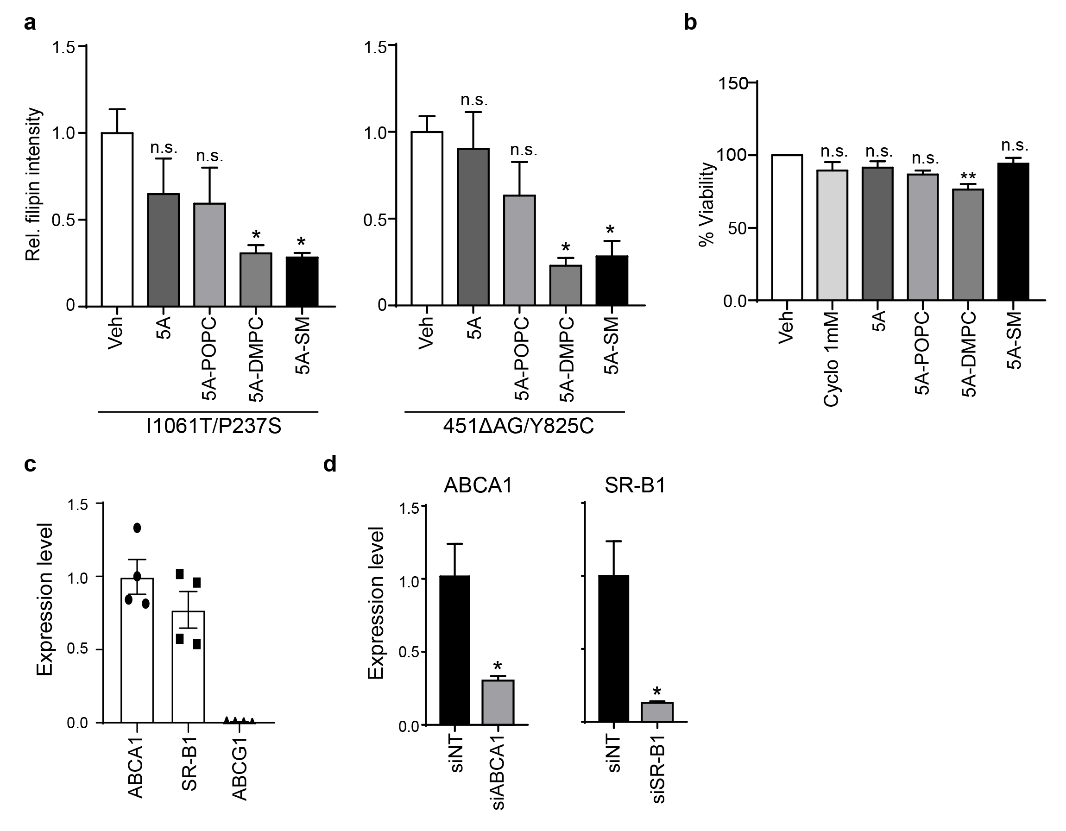
**

**Figure Supplementary 1. sHDLs rescue cholesterol storage in Niemann-Pick C patient fibroblasts.**

(a) Primary human fibroblasts with compound heterozygous mutations in NPC1, encoding I1061T/P237S or 451ΔAG/Y825C, were treated with vehicle (Veh) or 0.75 mg/ml of 5A peptide, 5A-POPC, 5A-DMPC, or 5A-SM for 48 hrs. Unesterified cholesterol was labeled with filipin and quantified.

(b) Viability of Niemann-Pick C patient (I1061T/I1061T) primary fibroblasts after 24-hour treatment with vehicle (Veh), 1 mM cyclodextrin (Cyclo), 0.75 mg/ml of 5A peptide, 5A-POPC, 5A-DMPC, or 5A-SM.

(c) qPCR was used to assess expression level of ABCA1, SR-B1, and ABCG1 mRNAs in NPC1 I1061T primary fibroblasts.

(d) NPC1 I1061T primary fibroblasts were treated with siRNAs against ABCA1 (siABCA1) or SR-B1 (siSR-B1), or with non-targeting (NT) siRNAs for 48 hrs. mRNA expression was analyzed by qPCR.

Data are mean ± s.e.m. from (a, d) three, (c) four or (b) seven independent experiments. (a, b) One-way ANOVA with Tukey posthoc test (F, df=(a) (Left) 4.015, 4; (Right) 6.603, 3; (b) 4.171, 5). (d) Student’s t-test (t=(ABCA1) 3.20, (SR-B1) 3.69). n.s., not significant, *p≤.0.05, **p≤.01.


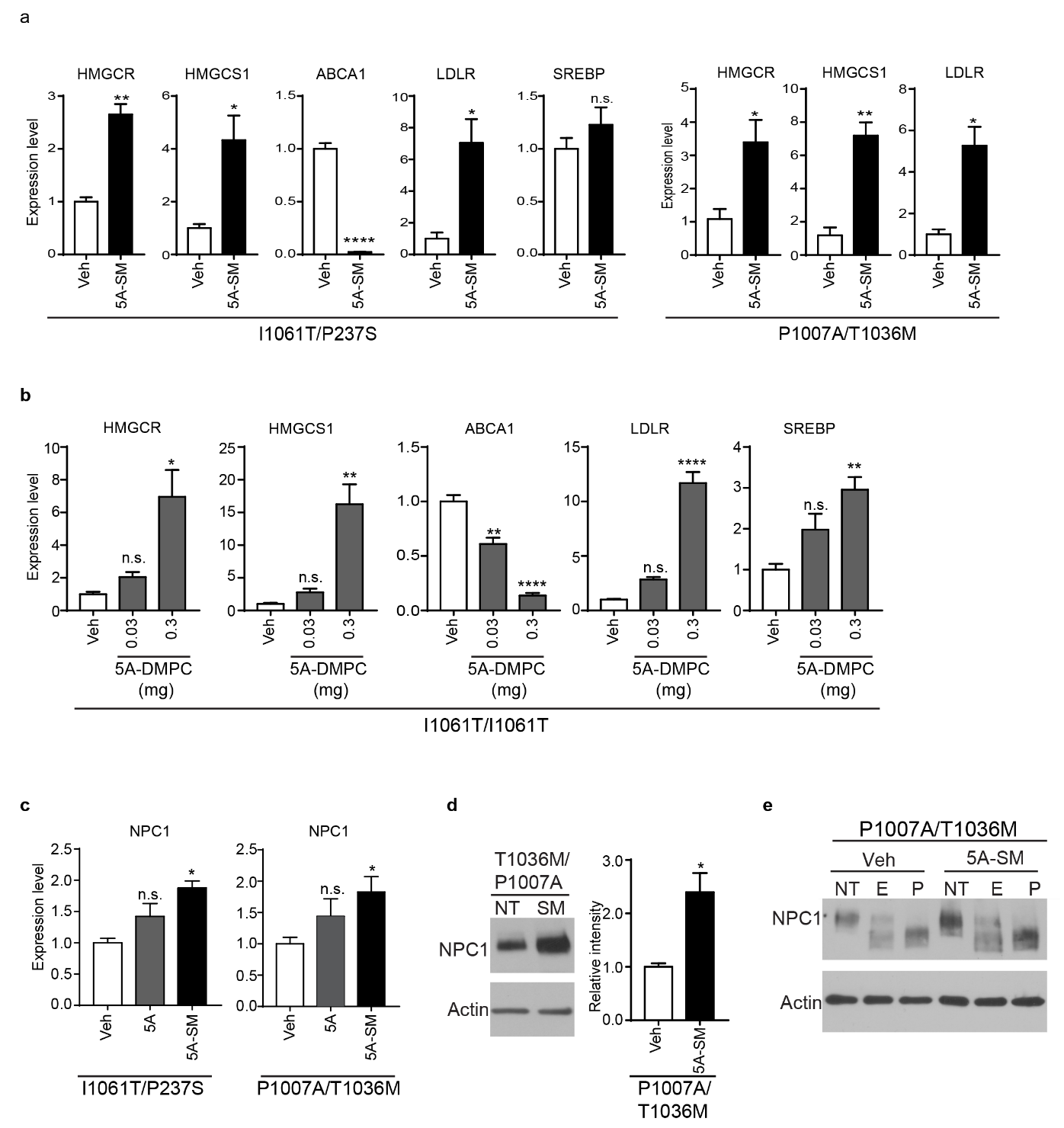


**Figure Supplementary 2. sHDLs induce the expression of cholesterol regulatory genes.**

(a) Primary human fibroblasts with (a, c) I1061T/P237S, P1007A/T1036M, or (b) I1061T/I1061T NPC1 alleles were treated with vehicle (Veh), (a, c) 0.75 mg/ml 5A-SM, or (b) increasing concentrations of 5A-DMPC for 48 hrs. mRNA expression of cholesterol regulatory genes was analyzed by qPCR.

(d, e) NPC1 protein levels from P1007A/T1036M fibroblasts treated with vehicle (Veh) or 5A-SM were analyzed by western blot for (d) total levels or (e) digested with endoglycosidase H (E), PNGaseF (P) or not treated (NT).

Data are mean ± s.e.m. from three independent experiments. (a, d) Student’s t-test (t=(a) I1061T/P237S (HMGCR) 5.71; (HMGCS1) 38.35; (ABCA1) 17.92; (LDLR) 3.91; (SREBP) 1.173; P1007A/T1036M (HMGCR) 3.31; (HMGCS1) 6.43; (LDLR) 4.53; (d) 4.329); (b, c) One-way ANOVA with Tukey posthoc test (F, df=(b) (HMGCR) 10.73, 2; (HMGCS1) 21.72, 2; (ABCA1) 75.05, 2; (LDLR) 89.24, 2; (SREBP) 10.7, 2; (c) (Left) 9.78, 2; (Right) 5.23, 2). n.s., not significant, *p≤.0.05, **p≤.01, ****p≤.0001.


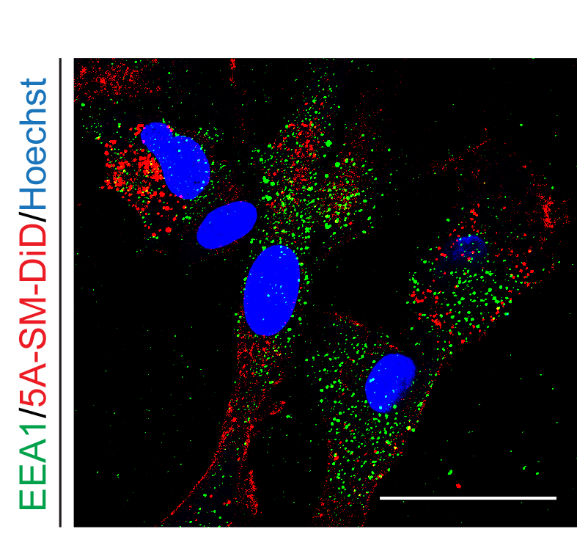


**Figure Supplementary 3. 5A-SM does not co-localize with EEA1 at two hours post treatment.** NPC1 I1061T fibroblasts were treated with 0.75 mg/ml 5A-SM-DiD (red) for two hours. Cells were fixed and stained for EEA1 (green) and nuclei (blue). Representative images from 2 hours post treatment. Scale bar = 50 µm.


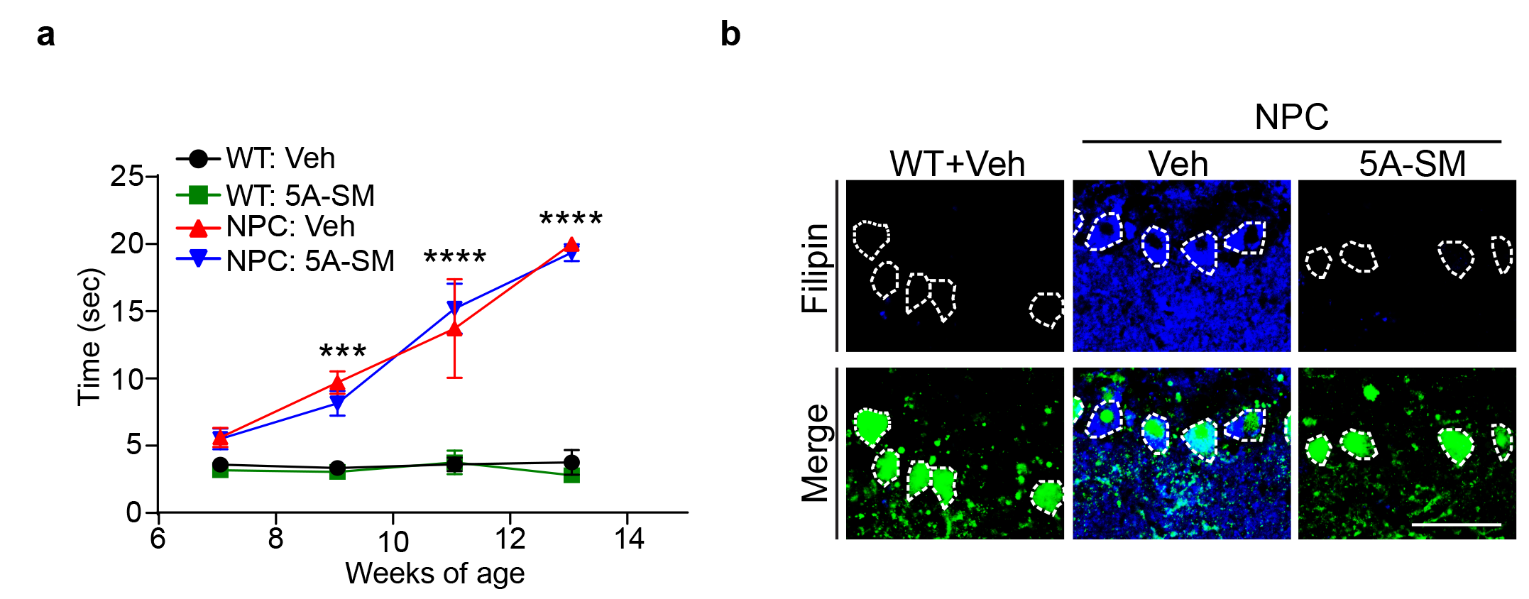


**Figure Supplementary 4. Effects of 5A-SM treatment.**

(a) Mice were treated i.p. with vehicle (Veh) or 100 mg/kg 5A-SM three times per week from 7 days to 13 weeks of age. Motor function was tested every other week from 7-13 weeks of age by balance beam. Data are mean ± s.e.m. from WT+Veh (n=6-10), WT + 5A-SM (n=5-6), NPC+Veh (n=3-5), NPC+5A-SM (n=4) mice. Two-way ANOVA with Bonferonni posthoc test (F, df= (52.46, 3). n.s., not significant, *p≤.0.05, **p≤.01, ***p≤.001, ****p≤.0001.

(b) Brain slices from 8 week old Npc1 *WT* or *I1061T* (NPC) mice were incubated with vehicle (Veh) or 5 mg/ml 5A-SM for four days. Purkinje cells were labeled with calbindin (green) and cholesterol with filipin (blue). Images were taken from lobules 3-6. Dashed lines outline Purkinje cell soma. Scale bar = 50 µm.


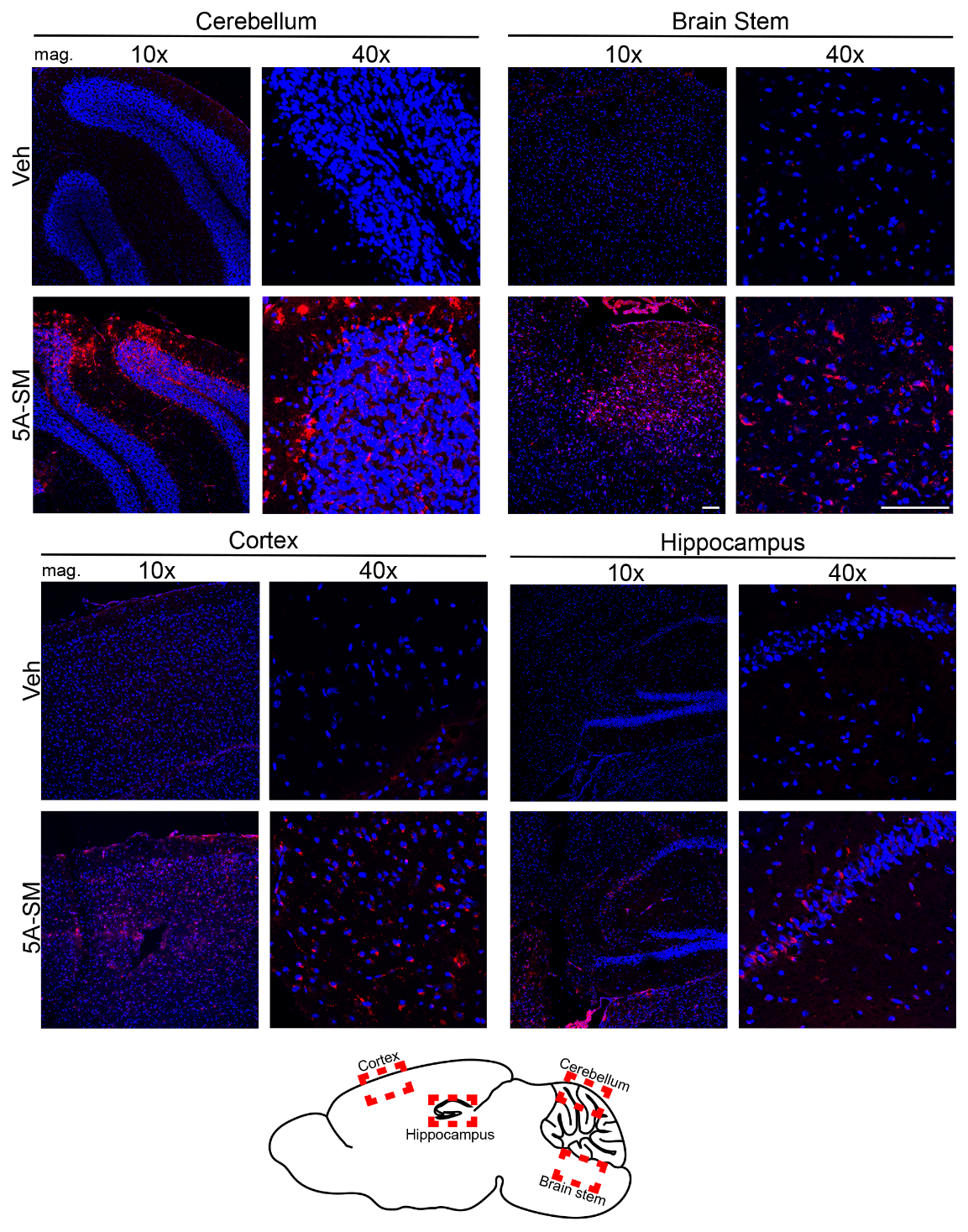


**Figure Supplementary 5. 5A-SM distribution in the brain after ICV injection.** Seven-week-old *Npc1* *I1061T* mice were injected ICV with vehicle (Veh) or 5A-SM-DiD. One-week post injection confocal microscopy was used to visualize distribution of DiD (red) and DNA (blue). Images of the cerebellum, brainstem, cortex, and hippocampus are shown (regions of images indicated below in the cartoon). Scale bars = 100 µm.


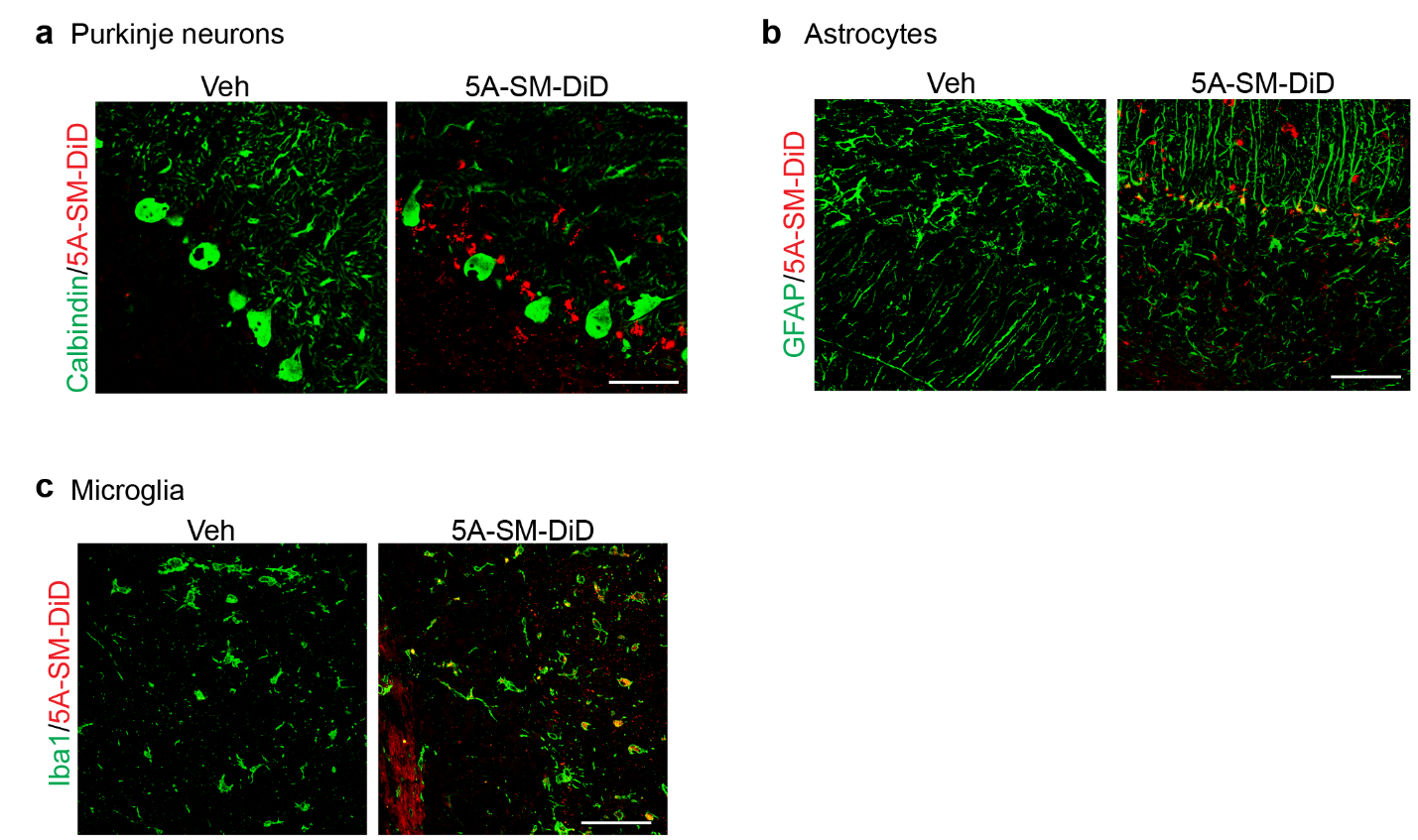


**Figure Supplementary 6. Cellular distribution of 5A-SM after ICV injection.**

Npc1 I1061T mice received intraventricular injections with vehicle (Veh) or 5A-SM-DiD. Confocal microscopy was used to analyze co-localization of DiD (red) in cerebellum with (a) Purkinje neurons (calbindin), (b) astrocytes (GFAP), or (c) microglia (Iba1). Scale bar = 50 µm.

**Uncropped blots:**


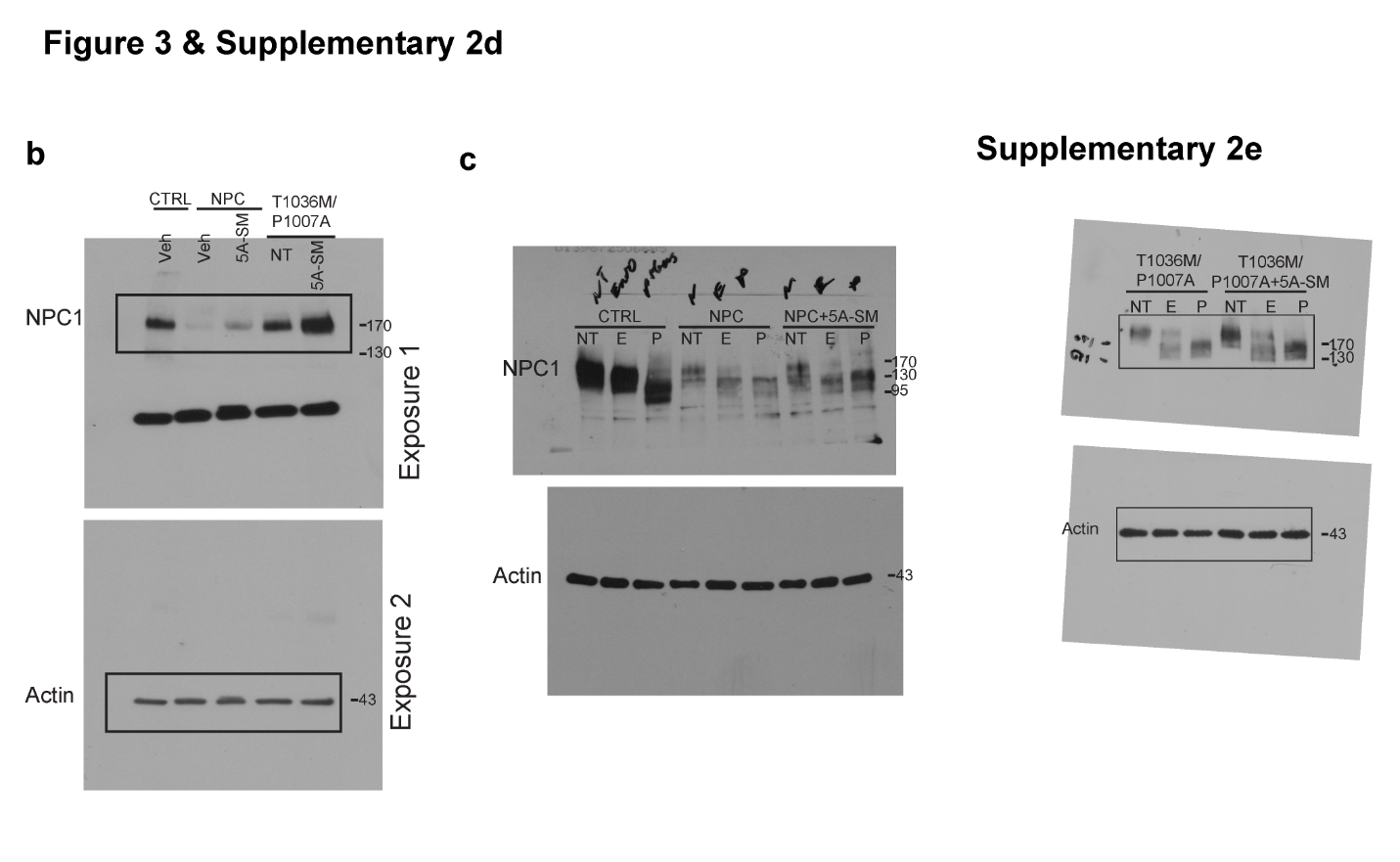

Supplement: Supplementary file 1 — Additional file 1: Table S1. Expected and actual peptide: lipid ratios. Figure S1. sHDLs rescue cholesterol storage in Niemann-Pick C patient fibroblasts. Figure S2. sHDLs induce the expression of cholesterol regulatory genes. Figure S3. 5A-SM-DiD does not co-localize with EEA1. Figure S4. Effects of 5A-SM treatment. Figure S5. 5A-SM distribution in the brain after ICV injection. Figure S6. Cellular distribution of 5A-SM after ICV injection. [file 12916_2019_1423_MOESM1_ESM.docx]
